# Unlocking the gasotransmitter: hydrogen sulfide as a multitarget regulator in ischemia–reperfusion injury

**DOI:** 10.4103/mgr.MEDGASRES-D-25-00100

**Published:** 2026-01-06

**Authors:** Ye Chen, Yulin Deng, Rong Tang, Shiqin Li, Zhigang Mei, Jinwen Ge

**Affiliations:** 1 Key Laboratory of Hunan Province for Integrated Traditional Chinese and Western Medicine on Prevention and Treatment of Cardio-Cerebral Diseases, College of Integrated Traditional Chinese and Western Medicine, Hunan University of Chinese Medicine, Changsha, Hunan Province, China; 2 Academy of Chinese Medical Sciences, Hunan University of Chinese Medicine, Changsha, Hunan Province, China; 3 Hunan Academy of Chinese Medicine, Changsha, Hunan Province, China

**Keywords:** apoptosis, autophagy, future application, gasotransmitter, hydrogen sulfide donors, hydrogen sulfide, inflammation, ischemia–reperfusion injury, oxidative stress, pharmacological strategies

## Abstract

Ischemia–reperfusion injury, a critical pathophysiological phenomenon in multiple organ systems, remains a formidable therapeutic challenge in clinical practice. As the third endogenously produced gaseous signaling molecule, hydrogen sulfide (H_2_S) has emerged as a pivotal regulator of diverse physiological processes and pathological cascades. Accumulating evidence indicates that H_2_S exerts cytoprotective effects against cerebral, cardiac, hepatic, renal, and pulmonary ischemia–reperfusion injuries through multifaceted mechanisms involving mitigation of inflammatory responses, suppression of oxidative stress, modulation of autophagic processes, and inhibition of apoptotic pathways. This comprehensive review systematically examines the endogenous biosynthesis and metabolic regulation of H_2_S, while elucidating the molecular mechanisms underlying its organ protective effects during ischemia–reperfusion injury. Particular emphasis is placed on the therapeutic potential of H_2_S synthase isoforms and bioactive metabolites in ischemic pathophysiology. Notably, recent advances in H_2_S pharmacology have catalyzed the development of novel H_2_S donors and slow-releasing compounds, including HSDF-NH_2_, S-allyl cysteine, S-propargyl cysteine, and S-(4-fluorobenzyl)-N-(3,4,5-trimethoxybenzoyl)-L-cysteine. These pharmacological innovations demonstrate enhanced tissue specificity and controlled release kinetics, paving the way for clinical translation of H_2_S-based therapeutics in ischemia–reperfusion injury management. Future research directions should focus on optimizing drug delivery systems and elucidating the spatiotemporal dynamics of H_2_S signaling in organ-specific ischemia–reperfusion pathologies.

## Introduction

Ischemia–reperfusion injury (IRI) refers to a pathological phenomenon where tissues or organs experience aggravated damage upon restored blood perfusion following ischemia, primarily due to reoxygenation-induced stress.^1^ This pathophysiological process typically manifests as a “two-phase assault”: The initial ischemic phase induces hypoxic metabolic disorders, while the subsequent reperfusion phase paradoxically exacerbates cellular structural and functional damage through complex mechanisms involving oxidative stress (OS), apoptosis, dysregulated autophagy, and inflammatory cascades.^2^ As a clinically prevalent pathological basis, IRI extensively occurs in diverse medical contexts including trauma management, interventional therapies, organ transplantation, and ischemic cardiovascular/cerebrovascular diseases. This prominence has positioned the exploration of effective intervention strategies as a critical focus in translational medicine research.

**Figure 1:**
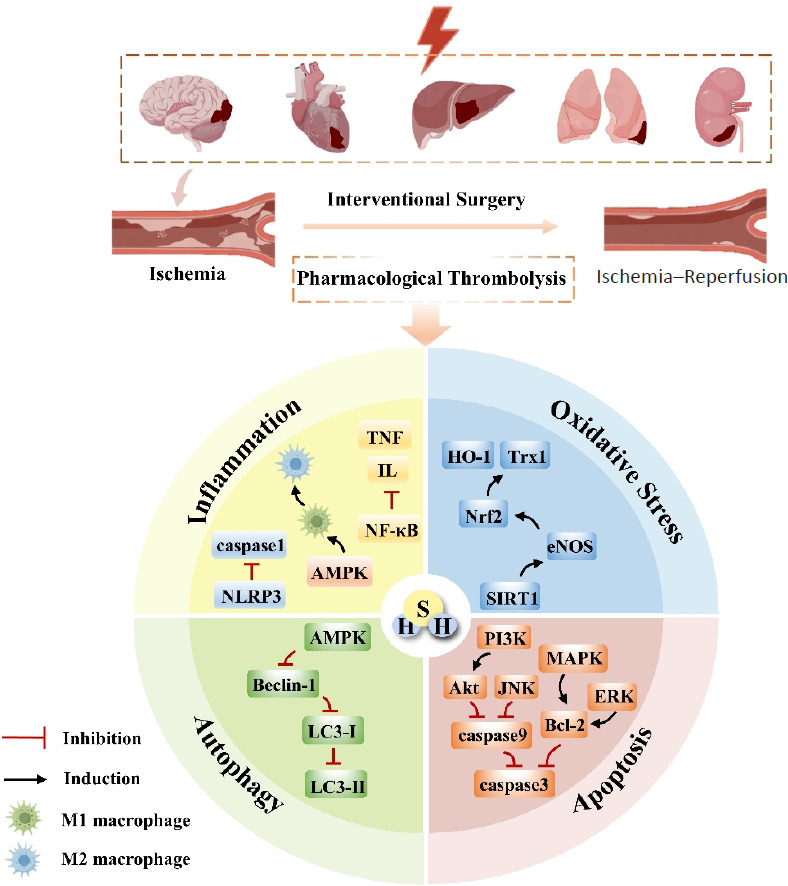
The role of H_2_S in organ ischemia–reperfusion injury. The application of H_2_S treatment after ischemia can alleviate ischemia-reperfusion injury of the brain, heart, kidney, liver and lung. Created with Microsoft PowerPoint 2021, some of the image materials are from Home for Researchers (www.home-for-researchers.com). Akt: Protein kinase B; AMPK: AMP-activated protein kinase; Bcl-2: B-cell lymphoma 2; eNOS: endothelial nitric oxide synthase; ERK: extracellular signal-regulated protein kinase; HO-1: hemoglobin oxygenase-1; IL: interleukin; JNK: c-Jun N-terminal kinase; LC3-I: light chain 3I LC3-II: light chain 3II; MAPK: mitogen-activated protein kinase; NF-κB: nuclear factor-κB; NLRP3: NLR family pyrin domain containing 3; Nrf2: nuclear factor erythroid 2-related 2; PI3K: phosphatidylinositol 3-kinase; SIRT1; sirtuin1; TNF: tumor necrosis factor; Trx1: thioredoxin-1.

Recent advances in gaseous signal transduction research have revealed that endogenous gaseous molecules nitric oxide (NO), carbon monoxide (CO), and hydrogen sulfide (H_2_S) exert synergistic regulatory effects in protection against through multi-pathway crosstalk mechanisms.^3^ Particularly, H_2_S has attracted significant attention due to its unique “double-edge sword” biological properties: As a toxic gas with pungent odor, concentrations exceeding 100 ppm can induce neurotoxic effects^4^; yet within physiological ranges, it exhibits crucial functions including neuroprotection and vasodilation regulation.^5^,^6^ Notably, as the third endogenous gasotransmitter, H_2_S leverages its lipophilic properties to freely penetrate bio-membranes, actively participating in vital physiological processes such as organ protection, metabolic regulation, and inflammatory modulation through direct modification of protein sulfhydryl groups or regulation of signaling pathways.^7^ With the continuous advancement of scientific research, the latest experimental data have confirmed that H_2_S exerts dual regulatory roles in living organisms: Participating in the maintenance of physiological homeostasis while also playing a pivotal role in the pathogenesis and progression of various diseases,^8^ and the protection against IRI in brain, heart, kidney, liver, and lung^9^,^10^,^11^,^12^,^13^ (**Figure 1**).

Although the therapeutic potential of H_2_S in IRI management is increasingly recognized, its precise molecular network remains uncharted. This review systematically elucidates the molecular regulatory mechanisms of H_2_S and its donors in IRI, with particular emphasis on deciphering the pharmacological foundations underlying their multi-dimensional organ-protective effects. The synthesis aims to provide theoretical frameworks and translational perspectives for developing novel therapeutic strategies. To achieve this goal, we conducted a comprehensive literature search via PubMed to identify relevant articles published over the past 35 years, with a focus on those published in the past 3 to 5 years. The search terms used included H_2_S, H_2_S donors, and IRI. The search results included original studies, review articles, and meta-analyses. We included high-quality literature that was directly relevant to the research topic and for which the full text was available.

## Generation and Metabolism of Hydrogen Sulfide

### Synthesis of hydrogen sulfide

**Figure 2:**
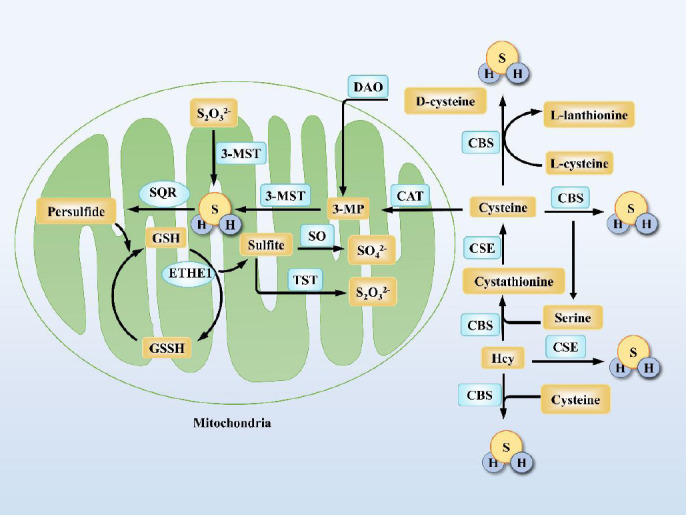
Mammals produce endogenous H_2_S through three enzymatic pathways: CBS, CSE, and 3-MST/CAT. Multiple enzymatic and non-enzymatic biochemical pathways are involved in the formation of sulfur metabolite. Sulfur catabolism leads to the metabolic end products sulfate and thiosulfate through the mitochondrial H_2_S oxidation pathway. Created with Microsoft PowerPoint 2021. 3-MP: 3-Mercaptopyruvate; 3-MST: 3-mercaptopyruvate sulfur transferase; CAT: cysteine aminotransferase; CBS: cystathionine β-synthase; CSE: cystathionine γ-lyase; DAO: D-aminoacid oxidase; H_2_S: hydrogen sulfide; Hcy: cysteine and homocysteine; S_2_O_3_^2−^: thiosulfate; SO_4_^2−^: sulfate; SO: sulfite oxidase; TST: thiosulfate sulfur transferase.

H_2_S in mammals is generated mainly through enzyme-catalyzed reactions and non-enzymatic pathways (**Figure 2**). In the enzymatic generation system, there are three major metabolic pathways mediated by four classes of enzymes: Cystathionine-β-synthase (CBS), cystathionine-γ-cleaving enzyme (CSE), 3-mercapto pyruvate sulfotransferase (3-MST), and cysteine aminotransferase (CAT). Among them, CBS and CSE are involved in H_2_S biosynthesis by catalyzing the metabolic reaction of L-cysteine with homocysteine (Hcy). Notably, L-cysteine, as a key metabolic precursor, plays a central substrate role in about 80% of endogenous H_2_S production in the body.^14^

CBS is the main enzyme that produces H_2_S,^15^ and mainly expressed in astrocytes.^16^ CBS catalyzes three reactions including the transformation of cysteine to serine and H_2_S, the polycondensation of cysteine and Hcy to produce cystathionine and H_2_S, and the binding of two cysteine molecules to produce H_2_S. While the enzymatic kinetics of cysteine-mediated H_2_S production are less efficient than the canonical serine and Hcy condensation, simulations under physiological conditions demonstrate that cysteine can competitively inhibit serine binding to CBS, resulting in significant H_2_S generation. The predominant pathway for H_2_S production involves cysteine condensation with Hcy. When cysteine abundance surpasses serine, and Hcy levels are limited, cysteine desulfurization becomes the primary mechanism. Tissues with elevated serine concentrations favor thioether synthesis over H_2_S formation, whereas lower serine levels enhance H_2_S generation.^17^ CBS activity is regulated by glutathione (GSH).^18^ In addition, the activity of CBS is affected when heme located at the amino terminus of CBS binds to CO and NO.^19^

The biological distribution of CSE, as a key component of the H_2_S biosynthetic system, shows remarkable tissue specificity: Immunohistochemical analysis confirmed that the enzyme is mainly localized in the cytoplasmic compartments of liver parenchymal cells, renal tubular epithelial cells, the vascular system, and reproductive organs.^20^ From the metabolic substrate perspective, L-cysteine not only serves as a core reactant for CBS-catalyzed H_2_S generation, but also can be converted to H_2_S precursors via the CSE-dependent transculturation pathway.^21^ Notably, in the co-catalytic network formed by CBS and CSE, CBS and CSE can convert two molecules of L-cysteine to H_2_S and the by-product L-lanthionine via a β-substitution reaction mechanism, a process involving the targeted transfer of sulfhydryl groups and specific changes in the α-carbon stereo conformation.^22^ Analysis of the biochemical mechanisms suggests that CSE and CBS jointly regulate the spatiotemporal-specific generation of intracellular H_2_S through the formation of a dynamic network of enzymatic reactions, in which CSE dominates H_2_S metabolism in peripheral tissues, whereas CBS plays a central role in the maintenance of H_2_S homeostasis in the central nervous system (CNS).

There exists a third metabolic axis of mitochondrial endogenous H_2_S biosynthesis independent of CBS and CSE, which consists of a pathway co-catalyzed by CAT and 3-MST. Proteomic analysis showed that 3-MST expression showed significant organ distribution heterogeneity: High expression in brain tissue (especially in neuronal synaptic regions), cardiomyocyte mitochondria, alveolar epithelial cells, intestinal mucosal crypt regions, and pancreatic alveolar cells, whereas relatively low levels were found in the central venous region of the hepatic lobule, renal medulla, and the bottom of the colonic crypts.^23^ 3-MST generates H_2_S from thiosulfate.^24^ In addition, a new mechanism for H_2_S production by 3-MST from D-cystine has been identified. After absorption of D-cysteine into the bloodstream via the gastrointestinal tract, it is metabolized by D-amino acid oxidase to 3-mercaptopyruvate, which is the substrate for the production of H_2_S by 3-MST.^25^ Notably, this D-cystine-dependent H_2_S generation pathway has a clear spatial specificity and is mainly enriched in the mitochondria of the cerebellar Purkinje cell layer and the mitochondrial endomembrane system of the proximal tubular epithelial cells of the kidney.^26^

A multidimensional chemical mechanism exists for the non-enzymatic biosynthesis of H_2_S: First, in the sugar metabolism system, glucose can release H_2_S through redox coupling reactions via metabolic scenarios such as glycolytic pathway intermediates or the nicotinamide adenine dinucleotide phosphate (NADPH) oxidase-mediated pentose phosphate bypass (which generates gluconose 6-phosphate); Second, when reducing monosaccharides are combined with sulfur-containing amino acids (Hcy, cysteine) undergo nucleophilic substitution reactions, gaseous sulfide complexes (mixed systems of methanethiol-dimethyl sulphide and H_2_S) can be formed. Emerging research demonstrates that within sulfur transfer reaction systems, GSH and elemental sulfur can generate biologically active H_2_S through non-enzymatic reactions mediated by thiol-driven reductive pathways. This discovery substantially expands the current understanding of endogenous H_2_S biosynthesis pathways in living organisms.^27^ Yang et al.^28^ found that non-enzymatic H_2_S production in a normal environment, using various H_2_S and metabolite assays, is partially dependent on cysteine as a substrate and is catalyzed by the synergistic activities of vitamin B6 and iron. Simultaneously, the amino group of cysteine initiates a nucleophilic attack on the pyridoxal (phosphate) moiety, forming the primary cysteine aldimine intermediate. Free or heme-bound iron ions subsequently drive the conversion to cysteine quinone, mediating sulfhydryl elimination while hydrolyzing the desulfurized aldimine back to pyridoxal (phosphate). This cascade culminates in the production of pyruvate, ammonia, and H_2_S. These findings indicate that sulfur-containing amino acids can produce H_2_S non-enzymatically. Notably, in addition to serving as a specific substrate for the enzymatic reaction of 3-MST, the thioester bond of 3-mercaptopyruvate can be spontaneously ruptured at the physiological sulfhydryl-disulfide bond redox potential, releasing H_2_S via a free radical-mediated desulfurization process.^29^

### Catabolism of hydrogen sulfide

Metabolic removal of H_2_S via the detoxification pathway is essential for maintaining normal physiological homeostasis of sulfide and its metabolites. Its catabolic properties via the mitochondrial respiratory oxidative pathway make it an important respiratory fuel for the body.^30^ Mitochondrial membranes harbor sulfur-quinone oxidoreductase, catalyzing electron transfer between H_2_S and coenzyme Q to produce the intermediate persulfide.^31^ This persulfide is subsequently transferred to GSH and acceptors, forming GSH peroxysulfide.^32^ GSH is further oxidized by the iron-dependent persulfide dioxygenase ethylmalonic encephalopathy protein 1 to produce sulfite, which is then converted to sulfate by sulfite oxidase. Additionally, sulfite reacts with GSH to produce GSH and thiosulfate, the latter being a final product of the pathway alongside sulfate. Thiosulfate sulfotransferase catalyzes the *in vitro* synthesis of these end products.^33^ A secondary mechanism for H_2_S catabolism involves its methylation to methyl mercaptan and dimethyl sulfide by cytoplasmic thiol S-methyltransferases. Furthermore, H_2_S interacts with methemoglobin to form sulfur–methemoglobin, proposed as a potential biomarker for plasma H_2_S consumption.^34^ In addition, tissue H_2_S can be stored as thioalkane sulfur (containing polysulfide and persulfide forms), which releases reactive H_2_S molecules under specific physiological conditions.^35^

## Protective Mechanisms of Hydrogen Sulfide in Ischemia–Reperfusion Injury

### Mechanisms associated with hydrogen sulfide

**Figure 3:**
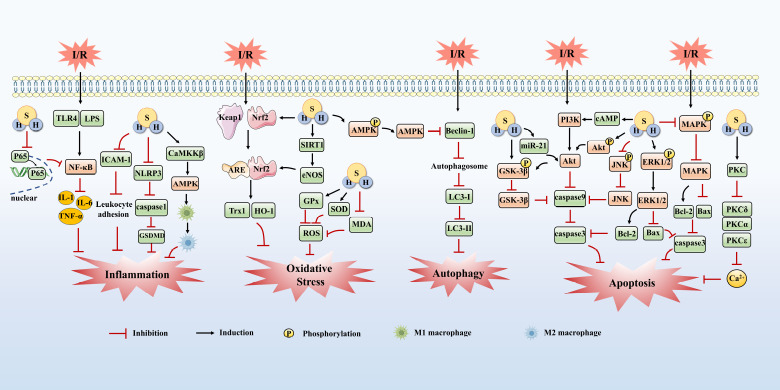
Mechanism of H_2_S on ischemia–reperfusion injury. The figure shows the protective mechanism of H_2_S in ischemia-reperfusion and some biological effects, including anti-oxidative stress, anti-inflammatory, anti-apoptosis, and anti-autophagy. Created with Microsoft PowerPoint 2021, some of the image materials are from Home for Researchers (www.home-for-researchers.com). Akt: Protein kinase B; AMPK: AMP-activated protein kinase; ARE: antioxidant response elements; Bax: Bcl-2 associated X protein; Bcl-2: B-cell lymphoma 2; cAMP: cycloadenylic acid 3’,5’-monophosphate; eNOS: endothelial nitric oxide synthase; ERK1/2: extracellular signal-regulated kinase 1/2; GPx: glutathione peroxidase; GSDMD: gasdermin D; GSK-3β: glycogen synthase kinase-3 β; H_2_S: hydrogen sulfide; HO-1: heme oxygenase-1; ICAM-1: intercellular cell adhesion molecule 1; IL-1: interleukin 1; IL-6: interleukin 6; JNK: c-Jun N-terminal kinase; Keap1: kelch-like ECH-associated protein 1; LC3-I: light chain 3I; LC3-II: light chain 3II; LPS: lipopolysaccharide; MAPK: mitogen-activated protein kinase; MDA: malondialdehyde; NF-κB: nuclear factor κB; Nrf2: nuclear factor erythroid 2-related 2; PI3K: phosphatidylinositol 3-kinase; PKC: protein kinase C; PKCα: protein kinase C α; PKCδ: protein kinase C δ; PKCε: protein kinase C ε; ROS: reactive oxygen species; SIRT1: sirtuin 1; SOD: superoxide dismutase; TLR4: Toll-like receptor-4; TNF-α: tumor necrosis factor α; Trx1: thioredoxin-1.

Similar to the established roles of NO and CO, H_2_S has recently emerged as a critical mediator in IRI. Accumulating evidence demonstrates that both pharmacological modulation of endogenous H_2_S biosynthesis and therapeutic supplementation of exogenous H_2_S exert broad-spectrum cytoprotective effects across diverse organ systems, mediated through distinct yet interconnected signaling pathways. This review systematically delineates the organ-specific protective actions of H_2_S in experimental IRI models and elucidates the mechanistic basis of its cellular safeguards, providing a comprehensive framework for understanding its therapeutic potential (**Figure 3**).

#### Hydrogen sulfide with nuclear factor-κB-related pathways

The nuclear factor-κB (NF-κB) signaling system consists of a canonical pathway and a non-canonical pathway. The canonical pathway is activated by a variety of extracellular stimuli, which triggers the ubiquitination degradation of inhibitor of NF-κB (IκB) proteins by inducing the phosphorylation of IκB kinase complex, leading to the rapid nuclear translocation of NF-κB dimer, which mainly regulates the expression of pro-inflammatory cytokines, such as tumor necrosis factor-α (TNF-α) and interleukin (IL)-6, and constitutes the core regulatory network of the intrinsic immune response. In contrast, the activation of the non-classical pathway is dependent on the ligand binding of specific TNF receptor superfamily members, which is mainly implicated in biological processes such as lymphoid organ development and adaptive immunomodulation.^36^

The inflammatory process following acute ischemic stroke (IS) is divided into three stages. These include (1) the acute stage, a series of pathological responses caused by insufficient tissue blood supply that activates microglia and promotes neutrophils to enter the blood–brain barrier (BBB)^37^; (2) the subacute stage, inflammation subsided; and (3) the late stage, involving nerve inflammation within damaged tissue (astrocytes and microglia, etc.), repair processes, and glial scar formation, ultimately contributing to a poor prognosis.^38^ In this process, microglia, astrocytes and peripherally infiltrated neutrophils/macrophages synergistically drive IRI through specific molecular mechanisms.^39^,^40^,^41^,^42^ Microglia, as resident immune sentinels in the brain parenchyma,^43^ become activated upon exposure to lipopolysaccharide (LPS) through interaction with toll-like receptor 4 (TLR4) on their membranes following a stroke. Subsequently, the NF-κB pathway is activated and triggers the conversion of microglia to a pro-inflammatory phenotype (M1).^44^,^45^ Experimental evidence suggests that exogenous H_2_S, when intervened at a concentration of 80 ppm in the early stage of reperfusion (within 1.5 hours), can inhibit NF-κB nuclear translocation by blocking IκBα phosphorylation, which in turn down-regulates the expression of inflammatory factors, such as TNF-α, interleukin 1β (IL-1β), and IL-6, and effectively curbs microglial M1 phenotype transformation and neuronal toxicity. It is worth noting that the inhibitory effect of H_2_S on the NF-κB pathway presents a dose-time double-dependent feature, and its specific molecular targets remain to be elucidated.^16^,^46^,^47^,^48^ At the level of transcriptional regulation, NF-κB activation requires key steps such as cytoplasmic inhibitor dissociation, phosphorylation of the Ser536 site of the p65 subunit and exposure of nuclear localization sequences.^49^ A study showed that in astrocytes, H_2_S inhibits the NF-κB pathway by reducing NF-κB p65 nuclear allosterism.^50^ In addition, the regulation of chemokine network by H_2_S is also pathologically important, monocyte chemotactic protein 1, another pro-inflammatory mediator, accumulates in large numbers around cerebral vascular cells after cerebral IRI (CIRI), promoting monocyte recruitment and the expression of adhesion molecules,^51^ whereas H_2_S can attenuate inflammation by preventing monocyte chemotactic protein 1 aggregation.^52^

Ustunova et al.^53^ employed the Langendorff *ex vivo* heart perfusion model to systematically elucidate the protective mechanisms of H_2_S against myocardial IRI (MIRI). Experimental data demonstrate that intervention with the H_2_S donor compound sodium hydrosulfide (NaHS) significantly inhibits the release of myocardial injury biomarkers creatine kinase-MB isoenzyme and lactate dehydrogenase, while concurrently enhancing the activity of the endogenous antioxidant enzyme GSH peroxidase (GPx). Histopathological analysis further confirmed that NaHS was effective in attenuating ultrastructural damage such as myocardial fiber breaks and mitochondrial vacuolization. Mechanistically, western blot analysis revealed a significant reduction in nuclear expression of the NF-κB p65 subunit in NaHS-treated cardiomyocytes compared to control groups, demonstrating that H_2_S exerts cardioprotective effects against IRI by inhibiting the nuclear translocation process of the NF-κB signaling pathway.^54^ Studies have revealed that H_2_S has a concentration-dependent biphasic regulation of the NF-κB signaling pathway. It was observed in LPS-treated mouse macrophages that low doses of NaHS decreased NF-κB activity, whereas high doses of NaHS showed the opposite effect, not only increasing NF-κB activity but also promoting the release of pro-inflammatory factors.^55^ The aberrant upregulation of intercellular adhesion molecule-1 in endothelial cells constitutes a pivotal priming event for inflammatory cascade activation.^56^ NF-κB can activate intercellular adhesion molecule-1, which induces leukocyte adhesion, rolling, and then infiltration into target areas that induce inflammatory processes. A study demonstrated that H_2_S mediates anti-inflammatory effects in both normotensive rats and L-arginine methyl ester-induced hypertensive models by inhibiting the activation of NF-κB and downregulating the expression of intercellular adhesion molecule-1.^57^

#### Hydrogen sulfide with adenosine 5’-monophosphate-activated protein kinase-related pathways

As a central orchestrator of cellular energy homeostasis, adenosine 5′-monophosphate-activated protein kinase (AMPK) serves as a molecular sensor capable of detecting intracellular energy perturbations and becomes potently activated during metabolic stress, particularly under pathophysiological conditions such as hypoxia.^58^ Emerging evidence reveals that AMPK exerts cytoprotective effects in IRI through a dual regulatory mechanism: By simultaneously stimulating adenosine triphosphate (ATP)-producing catabolic pathways and suppressing ATP-depleting anabolic processes. This coordinated metabolic modulation maintains cellular energy equilibrium during ischemic challenges, while ultimately attenuating IRI-associated cellular damage through enhanced bioenergetic stability.^59^ Emerging evidence delineates that H_2_S exerts anti-inflammatory effects on microglia through coordinated activation of the calcium/calmodulin dependent protein kinase kinase β-AMPK signaling axis.^60^ Zhang et al.^61^ elucidated the H_2_S-mediated neuroprotective mechanism wherein AMPK activation facilitates phenotypic switching from pro-inflammatory M1 to anti-inflammatory M2 microglia under ischemic conditions. Their experimental models demonstrated significant downregulation of AMPK phosphorylation in ischemic microglia, a deficit that was effectively rescued by CBS overexpression-mediated H_2_S supplementation. Crucially, pharmacological inhibition of AMPK completely abolished H_2_S-induced M2 polarization, thereby confirming the pivotal role of this kinase in mediating phenotypic transition. This regulatory cascade was further substantiated by observations that targeted inhibition of calcium/calmodulin dependent protein kinase kinase β, the upstream activator of AMPK, similarly disrupted microglial M2 polarization, establishing the calcium/calmodulin dependent protein kinase kinase β-AMPK pathway as the principal molecular conduit for H_2_S-mediated immunomodulation.^60^

In a mechanistic study, Xie and colleagues^62^ elucidated the cardioprotective effects of H_2_S through AMPK-mediated regulation of MIRI. Employing a rat model of cardiac IRI with pharmacological interventions using the H_2_S donor 5-(4-methoxyphenyl)-3H-1,2-dithiole-3-thione (ADT) and AMPK-specific inhibitor Compound C, the researchers conducted a comprehensive analysis of AMPK activation dynamics and subsequent autophagic modulation. Key findings demonstrated that ADT administration significantly downregulated autophagy-related biomarkers (light chain 3II (LC3-II)/light chain 3I (LC3-I) ratio, Beclin-1, and P62) while upregulating lysosomal membrane protein lysosome-associated membrane protein-2 in a dose-dependent manner. Crucially, Compound C pretreatment completely abrogated ADT-mediated restoration of autophagic flux post-IRI. Mechanistic interrogation revealed that H_2_S enhances autophagic degradation efficiency via AMPK pathway activation, thereby mitigating mitochondrial and sarcoplasmic reticulum damage in cardiomyocytes. These findings not only delineate the H_2_S-AMPK axis as a critical regulator of cardiac autophagy homeostasis, but also pave the way for developing novel therapeutic strategies targeting this pathway in ischemic heart disease.

#### Hydrogen sulfide with NLR family pyrin domain containing 3-related pathways

As important pattern recognition receptors of the intrinsic immune system, members of the NLR family containing the pyrroline structural domain (represented by NLR family pyrin domain containing 3 (NLRP3)) have the structural feature of self-assembling to form typical multimeric protein complexes (inflammasomes). The oligomerization of this complex (usually in hexameric or heptameric conformation) constitutes a central molecular event in the amplification of the inflammatory signaling cascade, and its function is mainly dependent on the recruitment and activation of pro-caspase-1. Specifically, inflammatory vesicles induce self-shear activation of pro-caspase-1 through spatial site-blocking effects to generate catalytically active caspase-1 heterodimers. This activation process triggers the proteolytic maturation of IL precursors (pro-IL-1β and pro-IL-18), which in turn initiates cascading inflammatory signaling through the release of biologically active cytokines. This highly conserved molecular mechanism plays a dual regulatory role in host defense and pathological injury processes.^63^ The NLRP3 inflammasome is a multiprotein complex composed of NLRP3, apoptosis associated speck like protein (ASC) and pro-caspase-1. Upon activation, the NLRP3 inflammasome cleaves pro-caspase-1 into its active form, caspase-1, which subsequently mediates the maturation and secretion of pro-inflammatory cytokines, including IL-1β and IL-18, thereby playing a pivotal role in the regulation of inflammatory responses.^64^ Yang’s team^65^ elucidated the underlying mechanisms by establishing a rat cerebral ischemia–reperfusion (I/R) model. Their findings revealed that pretreatment with the H_2_S donor NaHS significantly ameliorated neurological deficit scores. Mechanistically, NaHS administration effectively suppressed NLRP3 inflammasome oligomerization and subsequent proteolytic activation of caspase-1, thereby impeding NLRP3 inflammasome assembly. This intervention led to a marked reduction in pro-inflammatory cytokine levels, including IL-1β and IL-18. Concurrently, NaHS downregulated critical downstream effectors of pyroptosis and apoptosis, notably gasdermin D (GSDMD).^65^ This study systematically revealed that H_2_S inhibits the formation of neuroinflammatory microenvironment by blocking the NLRP3/caspase-1/GSDMD signaling pathway, which in turn attenuates BBB disruption and neuronal programmed death, and highlighted the multi-targeted regulatory properties of H_2_S in cerebrovascular diseases. It is noteworthy that in retinal IRI models, the core regulatory factors of the pyroptosis activation pathway (NLRP3/GSDMD) were observed to be significantly upregulated. Application of voltage-dependent anion channel 1 oligomerization inhibitors effectively suppressed this aberrant activation and substantially ameliorated retinal tissue damage.^66^ However, since the current studies on H_2_S in the eye are mainly focused on CBS,^8^ there is current evidence remains insufficient to conclusively demonstrate whether H_2_S exerts its effects on retinopathy through the modulation of pyroptosis mechanisms. Elucidating this potential regulatory pathway may not only provide novel research directions for exploring therapeutic targets in retinal diseases but also potentially catalyze the development of innovative intervention strategies based on gasotransmitter-mediated regulation.

The interaction between H_2_S and NLRP3-related signaling pathways after IRI has been described. Sun’s team^67^ developed a novel slow-release H_2_S delivery system with mesoporous silica nanoparticles loaded with diallyl-trisulfide (DATS) for the model of MIRI, which exerts a cardioprotective effect through the precise regulation of the TLR4/NLRP3/caspase-1 signaling axis. A mechanistic study confirmed that mesoporous silica nanoparticles loaded with DATS dose-dependently inhibited TLR4 receptor activation, blocked the assembly of NLRP3 inflammatory vesicles, and significantly reduced the protein hydrolysis activity of caspase-1 in injured myocardial tissues.^67^ A finding that is mechanistically corroborated with Shibuya’s team’s conclusion on the modulation of intrinsic immune signaling pathways by H_2_S.^68^,^69^ Notably, the abnormal activation of NLRP3 inflammatory vesicles is a key molecular event in renal I/R pathology, leading to acute kidney injury (AKI) and significantly affecting the clinical prognosis of patients. Ni’s group^70^ constructed I/R models, including the hypoxia-reoxygenation model of rat renal tubular epithelial cells and the renal artery clamping model. They detected the expression of NLRP3 and ASC in both *in vivo* and *in vitro* models. The results showed that hypoxia/reoxygenation treatment significantly promoted the expression of NLRP3 and ASC, while the expression of NLRP3 and ASC was inhibited after NaHS treatment. It was clarified that the exogenous H_2_S donor NaHS can effectively inhibit the activity of NLRP3 inflammatory vesicles. Moreover, the high expression of caspase-1, GSDMD, IL-1β and IL-18 after I/R was reversed by NaHS. These groundbreaking findings systematically revealed the pervasive mechanism of H_2_S in attenuating organ IRI through targeted inhibition of the NLRP3/caspase-1 signaling cascade, providing new ideas for the development of precise organ protection strategies.

#### Hydrogen sulfide with phosphatidylinositol 3-kinase/protein kinase B-related pathways

Phosphatidylinositol 3-kinase (PI3K) is a lipid kinase composed of three subunits,^71^ and its downstream protein kinase B (Akt) can regulate cell physiological activities.^72^ Notably, this signaling pathway is specifically activated under pathological stress conditions and regulates the activity of the apoptosis-associated molecule caspase-9 through phosphorylation modification.^73^ In recent years, it has been found that the novel H_2_S donor S-(4-chlorobenzyl)-N-(3,4,5-trimethoxybenzoyl)-L-cysteine (MTC) exhibits significantly better anti-apoptotic effects than the conventional donor at sub-micromolar concentrations (1 μM).^74^ In the molecular regulatory network of CIRI, 70-kDa heat shock protein (HSP70) family members are involved in the maintenance of protein homeostasis through their molecular chaperone functions, which are specifically involved in key processes such as reprocessing of misfolded proteins, correct folding of nascent polypeptide chains, and ubiquitination degradation of damaged proteins.^75^ Experimental studies have shown that exogenous H_2_S intervention significantly improved the pathological process in the mouse CIRI model: Compared with the control group, the pretreatment group exhibited a significant reduction in cerebral infarct volume, accompanied by decreased levels of pro-inflammatory cytokines (IL-6 and TNF-α), suppressed activity of the apoptotic marker caspase-3, and diminished malondialdehyde (MDA) levels, an OS-associated indicator. Notably, H_2_S treatment induced up-regulation of HSP70 expression in brain tissues, whereas a sudden decrease in HSP70 expression and deterioration of neurological deficit scores were observed when the pathway was blocked by nuclear factor erythroid 2-related factor 2 (*Nrf2*) gene silencing or the PI3K inhibitor LY294002.^76^ These findings systematically revealed that H_2_S regulates HSP70-mediated cytoprotective mechanisms through the PI3K/Akt/Nrf2 signaling axis. Further studies confirmed that the neuroprotective effect of H_2_S has a multipathway synergistic property. In the rat hippocampal oxygen glucose deprivation/reoxygenation model, H_2_S triggered the PI3K/Akt/phosphoprotein 70 ribosomal protein S6 kinase cascade response by activating the cyclic adenosine monophosphate second messenger system, in which the phosphoprotein 70 ribosomal protein S6 kinase phosphorylation level was elevated, significantly enhancing the anti-apoptotic ability of neurons. This mechanism, together with the upregulation of HSP70, constitutes the dual defense system of H_2_S against CIRI.^77^

In the cardioprotective signaling network, glycogen synthase kinase-3β (GSK-3β) plays a central role as a key molecular hub of the PI3K/Akt and Wnt pathways by regulating the mitochondria-dependent apoptotic pathway. Phosphorylation inactivation of its Ser9 site can block the mitochondrial translocation of the pro-apoptotic protein B-cell lymphoma 2 (Bcl-2) associated X (Bax) and reduce the apoptotic rate of cardiomyocytes.^78^,^79^ Post-ischemic conditioning is a pathway that provides calcium protection and exerts cardioprotective effects through dual regulatory mechanisms: (1) endogenous activation of the CSE/H_2_S system elevates H_2_S production in cardiac tissues and significantly alleviates calcium overload-induced mitochondrial dysfunction; and (2) exogenous H_2_S donor NaHS synergistically enhances the post-ischemic conditioning effect by activating PI3K/Akt signaling axis and inducing phosphorylation at the GSK-3β, synchronously inhibiting *Nrf2*/antioxidant response element (ARE) pathway-mediated OS.^80^ Molecular mechanism studies have shown that serum-and glucocorticoid-inducible kinase 1, as a key downstream effector molecule of the PI3K signaling pathway, is involved in cellular homeostasis regulation through phosphorylation activation.^81^ In Jiang et al.’s study,^82^ PI3K inhibition using LY294002 or serum-and glucocorticoid-inducible kinase 1 knockdown enhanced autophagy while diminishing H_2_S’s anti-autophagic and protective effects, whereas GSK-3β inhibition using GSK-3β inhibitor XII had the opposite effect, suggesting that H_2_S inhibition via the PI3K/serum-and glucocorticoid-inducible kinase 1/GSK-3β pathway protects hypoxic/reoxygenated rat myocardial cells.

Upon systematic combing of existing studies, it was found that miR-21 plays biological roles in the liver mainly through the regulation of Akt signaling pathway and tensin homologue (PTEN).^83^ Lu et al.^84^ found that the increase of exogenous H_2_S concentration gradient could significantly up-regulate the expression level of miR-21, and the molecule works through a dual mechanism of action: On the one hand, by inhibiting phosphatase activity and down-regulation of PTEN expression, promoting the activation of Akt signaling pathway; on the other hand, it enhanced the phosphorylation modification of GSK-3β, which in turn inhibited its activity, and ultimately achieved the negative regulation of caspase-9/3 apoptotic pathway. Together, these molecular mechanisms explain the protective mechanism of H_2_S in alleviating hepatic IRI (HIRI) through the miR-21/Akt pathway axis.^85^ Molecular mechanism studies further revealed that miR-21 also reduces the expression of p38 mitogen-activated protein kinase (MAPK) protein, which in turn inhibits the activation of caspase-3 through the PTEN/Akt pathway.^86^

Current research reveals that autophagy exhibits dynamic activation characteristics during lung I/R pathology. Notably, activation of autophagic streams was shown to enhance lung tissue defense against IRI in an LPS-induced acute lung injury (ALI) model.^87^ Molecular mechanistic studies have shown that inhibition of PI3K/Akt signaling pathway activity and its mediated aberrant activation of autophagy are important pathological features in the development of ALI.^88^ Targeting this regulatory network, H_2_S can effectively inhibit autophagy overactivation by activating the PI3K/Akt/mammalian target of rapamycin (mTOR) signaling axis, which in turn attenuates LPS-induced structural damage in lung tissue.^87^ This protective effect was also validated in a hypertoxic ALI model, where enhanced PI3K/Akt signaling significantly improved alveolar epithelial barrier function.^89^ The research team further constructed the molecular regulatory network of miR-21 and TLR4, and found that miR-21 significantly reduced the level of inflammatory factor storm by directly inhibiting TLR4 expression or blocking NF-κB signaling in the LPS-ALI model.^90^ It is noteworthy that although H_2_S exhibits autophagy inhibitory properties in ALI, its autophagy regulation mechanism in the microenvironment of lung IRI (LIRI) is still subject to academic controversy, especially the interplay between autophagic flow dynamics and mitochondrial quality control needs to be resolved in depth.

#### Hydrogen sulfide with nuclear factor erythroid 2-related factor 2-related pathways

*Nrf2*, a pivotal transcriptional regulator of OS response, plays a critical regulatory role in IRI. Studies have demonstrated that *Nrf2* activates the antioxidant defense system and significantly upregulates the expression of antioxidant factors, including superoxide dismutase (SOD) and GPx, thereby effectively mitigating I/R-induced oxidative damage.^91^ In a rat model of cerebral I/R, the CBS/H_2_S system was shown to exert neuroprotective effects through a dual mechanism: On the one hand, it maintains calcium homeostasis by inhibiting reactive oxygen species (ROS)-activated calcium channels, and on the other hand, it significantly reduces apoptosis through activation of the *Nrf2*/hemoglobin oxygenase-1 (HO-1) signaling pathway.^92^ The regulation of *Nrf2* activity mainly involves two important pathways: The PI3K/Akt signaling pathway enhances *Nrf2* stability through phosphorylation modification, whereas the kelch-like epichlorohydrin-associated protein-1 (Keap1)-*Nrf2* axis regulates its nuclear translocation through protein interactions.^93^ In the physiological state, *Nrf2* and Keap1 form a complex anchored in the cytoplasm to maintain a basal level of antioxidant status.^94^ When cells experience OS, *Nrf2* separates from Keap1, initiating transcriptional activation of a variety of antioxidant genes.^95^ Within the cell nucleus, *Nrf2* initiates the transcriptional program of target genes by specifically binding to the ARE. Studies have confirmed that ARE, as a key cis-acting element, coordinately regulates the expression of antioxidant enzyme systems such as HO-1, thioredoxin and its reductase, forming a cascading antioxidant defense network.^96^ This multilevel regulatory mechanism makes *Nrf2* a core regulatory node for maintaining redox homeostasis, providing an important theoretical basis and therapeutic target for the prevention and treatment of IRI. Calvert’s team^97^ revealed the temporal regulatory features of the cardioprotective effects of H_2_S through animal experiments. The experimental data showed that significant nuclear translocation of *Nrf2* was observed in myocardial tissues 30 minutes after intraperitoneal injection of H_2_S, and its intranuclear concentration continued to maintain peak levels during the 2-hour observation period after administration. Notably, the antioxidant effect produced by this intervention showed a significant time-dependent profile after 24 hours of H_2_S exposure, Western blot assays showed upregulation of protein expression of thioredoxin 1 and HO-1, respectively, compared to the control group. This temporal gradient change from transcription factor activation to downstream effector protein expression suggests that H_2_S may exert a sustained cardioprotective effect through a cascade response mediated by the *Nrf2* signaling pathway.

In a study, the molecular mechanism of the synergistic cardioprotective effects of H_2_S and sevoflurane was systematically revealed by constructing a diabetic MIRI model. Innovative findings showed that H_2_S significantly enhanced the positive regulatory effect of sevoflurane on the *Nrf2*/HO-1 axis by activating the deacetylase activity of silencing regulatory protein 1 (Sirtuin1, SIRT1). Protein immunoblotting analysis showed that myocardial tissues of the combined intervention group showed elevated *Nrf2* nuclear translocation efficiency and a simultaneous increase in HO-1 protein expression, while NADPH oxidase expression was downregulated. Mechanistic studies further revealed that H_2_S elevated SIRT1 basal expression through epigenetic regulation, on the basis of which pretreatment with sevoflurane produced a synergistic effect. This cascade activation effect significantly improved mitochondrial functional homeostasis, as evidenced by a reduction in superoxide anion production; restoration of myocardial ATP content; and an increase in mitochondrial complex I/III activity. The study confirmed that H_2_S successfully reversed the attenuation of the cardioprotective effect of sevoflurane in the diabetic pathological state by strengthening the conduction efficacy of the SIRT1/*Nrf2* signaling pathway, which provided a new targeting strategy for the precise treatment of patients with diabetes mellitus combined with myocardial ischemia.^98^

A study on the mechanisms of OS regulation have shown that the *Nrf2*/HO-1 signaling pathway has a key regulatory role in diabetes-induced IRI.^99^ Notably, SIRT1 enhances the transcriptional activity and protein stability of *Nrf2* through deacetylation modification, thereby exerting dual protective effects with both antioxidant and anti-apoptotic properties.^100^ Further studies revealed that SIRT1 also co-localized with endothelial NO synthase (eNOS). Its deacetylation significantly enhances eNOS enzyme activity, whereas a decrease in SIRT1 activity leads to over acetylation of eNOS, which in turn triggers enzyme inactivation.^101^ The discovery of this regulatory mechanism provides a new perspective for the study of vascular lesions associated with metabolic diseases. Jiang et al.^102^ explored the alleviating effect of H_2_S pretreatment on diabetes-induced LIRI with the help of experiments by administering diabetic rats with morpholin-4-ium 4-methoxyphenyl (morpholino) phosphinodithioate (GYY4137) treatment prior to the implementation of hilar occlusion. GYY4137 treatment increased the expression of *Nrf2* in the nucleus. In contrast, inhibition of SIRT1 attenuated these effects, suggesting that SIRT1 is an upstream regulator of *Nrf2*/HO-1 signaling. GYY4137 also enhanced the phosphorylation of endothelial eNOS and decreased the acetylation of eNOS, whereas inhibition of SIRT1 reduced these effects. This study demonstrates that exogenous H_2_S activates the SIRT-1 pathway, which subsequently triggers antioxidant signaling cascades mediated by *Nrf2*/HO-1 and eNOS, while effectively attenuating pulmonary IRI during the warm ischemia phase through suppression of apoptotic pathways and inflammatory responses.

Studies have confirmed that the development of renal IRI (RIRI) is closely related to the *Nrf2* signaling pathway. Experimental data showed that *Nrf2* low-expressing mice exhibited higher susceptibility to ischemic kidney injury, and overactivation of this pathway effectively alleviated renal tubular injury in the early stages of RIRI.^103^ At the level of pathological mechanism, renal I/R was able to significantly activate the *Nrf2* signaling pathway and its downstream HO-1 protein expression. Notably, the activation level of NLRP3 inflammatory vesicles in renal tissues was significantly increased when *Nrf2* gene was knocked down, suggesting that *Nrf2* has an inhibitory effect on I/R-induced activation of the NLRP3 inflammatory pathway. A related study further found that NaHS could inhibit the activation of NLRP3 inflammatory vesicles by up-regulating *Nrf2* expression in a renal I/R model, but when the *Nrf2* gene was absent, this regulatory effect of NaHS was completely lost, suggesting that its protective effect was dependent on the *Nrf2* pathway.^104^ In terms of therapeutic intervention, the novel H_2_S donor GYY4137 demonstrated significant nephroprotective effects. Animal experiments demonstrated that the compound effectively ameliorated renal dysfunction and renal histoarchitectural damage in a RIRI model by enhancing antioxidant enzyme expression through activation of the *Nrf2* pathway. Further *in vitro* studies demonstrated that cells treated with GYY4137 exhibited significantly reduced MDA levels alongside markedly enhanced SOD activity, confirming its protective effects through antioxidant mechanisms.^105^

#### Hydrogen sulfide with mitogen-activated protein kinase-related pathways

The four major isoforms (extracellular signal-regulated kinase (ERK), c-Jun N-terminal kinase (JNK), p38 and ERK5) of the MAPK signaling pathway have key roles in apoptosis regulation, and they are involved in the molecular mechanisms of CIRI by mediating apoptosis-associated signaling cascade responses.^106^,^107^,^108^ Experimental studies have shown that middle cerebral artery occlusion (MCAO) triggers aberrant phosphorylation of MAPK family members, and specific inhibition of this pathway not only reduces the rate of neuronal apoptosis, but also significantly improves neurological recovery.^109^ Notably, p38 MAPK isoforms can accelerate programmed neuronal death under stress by regulating the transcription factor network.^110^ Targeting this mechanism, Liu et al.^111^ demonstrated that inhibition of the p38/JNK signaling axis and activation of the ERK1/2 pathway by pharmacological means could achieve differential regulation of the Bcl-2 protein family (Bax expression inhibition/Bcl-2 expression activation), which effectively blocked the apoptotic execution phase. Further studies revealed that exogenous H_2_S donors such as NaHS exhibited significant neuroprotective effects in an *in vitro* oxygen glucose deprivation/reoxygenation model by targeting and inhibiting p38 MAPK phosphorylation.^112^ This regulatory mechanism is cross-organ pervasive, and GYY4137, a slow-release H_2_S donor, demonstrated similar anti-apoptotic properties in a MIRI model.^113^

MAPK, ERK1/2 and PI3K can regulate the apoptotic pathway through dual mechanisms: One is through a cascade reaction with protein kinase C (PKC), and the other is through the inhibition of pro-apoptotic factors Bad and caspase-9 activity, which results in the negative regulation of the pro-apoptotic signaling pathway.^114^,^115^ Wei et al.^116^ in parsing the mechanism of neuroprotection by H_2_S found that lateral ventricle injection of PKC inhibitor Go6983 ((3-[1-[3-(dimethylamino)propyl]−5-methoxy-1H-indol-3-yl]−4-(1H-indol-3-yl)−1H-pyrrole-2,5-dione)) significantly elevated the expression level of aquaporin 4 and completely reversed the shrinking effect of H_2_S on the area of cerebral infarction in MCAO model. Mechanistic analysis indicated that H_2_S exerts neuroprotective functions by activating the PKC signaling axis. Notably, this regulation has a cross-organ protective effect in cardiomyocytes, H_2_S pretreatment can effectively reduce intracellular Ca^2+^ overload through specific activation of PKCα, PKCε, and PKCδ isoforms, and thus inhibit mitochondria-dependent apoptotic pathways.^117^

The ERK signaling pathway exhibits biphasic regulatory properties in the pathological process of IRI: On the one hand, it promotes tissue repair by activating endogenous neuroprotective mechanisms,^118^ and on the other hand, its key isoforms, ERK1/2, can inhibit mitochondria-dependent apoptotic pathways by up-regulating Bcl-2 expression.^119^ Studies have shown that IRI disrupts the balance of Bcl-2 family proteins, significantly upregulates the expression of the pro-apoptotic protein Bax, and subsequently induces the opening of mitochondrial permeability transition pores. This cascade further impairs Bcl-2-mediated retention of cytochrome c in mitochondria, ultimately leading to caspase-3 activation and triggering the apoptotic execution program.^120^ Therefore, by interfering with the Bcl-2/Bax protein ratio and inhibiting caspase-3 activity, mitochondrial membrane stability can be effectively maintained and neuronal survival after I/R can be increased.^121^

JNK1 serves as a key signaling hub to regulate HIRI, and its activity status directly affects the process of hepatocyte apoptosis. Consequently, regulating this pathway regulation of this pathway is expected to reduce liver injury.^122^ Tsung et al.^123^ reported activation of the JNK pathway and that inhibition of JNK activity attenuated apoptosis after liver I/R. Cheng et al.^124^ demonstrated that H_2_S preconditioning suppresses the activation of the JNK signaling pathway in HIRI, while the application of a JNK1-specific inhibitor further potentiates its protective effects against HIRI. Based on the above findings, researchers suggest that the hepatoprotective effect of H_2_S may stem from its mechanism of inhibiting apoptosis through regulation of the JNK signaling pathway. Furthermore, the study demonstrated that the NaHS can reduce programmed cell death in hepatocytes by modulating the expression levels of apoptosis-related proteins, a finding that provides additional evidence for the aforementioned mechanism.

#### Hydrogen sulfide with other proteins

OS is recognized as one of the key pathological mechanisms that induce IRI, which is essentially an imbalance of redox homeostasis in the organism.^125^ Under physiological conditions, the body precisely regulates ROS levels through endogenous antioxidant systems such as SOD and GPx. In this process, the dynamic balance between synthesis and scavenging of oxygen free radicals is maintained, effectively preserving the mitochondrial electron transport chain function and the integrity of the cell membrane structure.^126^ However, under hypoxic/ischemic pathological conditions, it has been demonstrated that the OS cascade significantly enhances the intensity of ROS generation.^127^ Margaill et al.^128^ identified four primary ROS sources: The mitochondrial respiratory chain, NADPH oxidase, cyclooxygenase-2-catalyzed arachidonic acid reaction, and xanthine oxidase-mediated xanthine and hypoxanthine production. This excessive OS can trigger multidimensional pathological damage, which is manifested as a complex of lipid peroxidation reactions, protein structural denaturation and loss of enzyme activity, impaired mitochondrial energy metabolism, and DNA strand breaks.^129^,^130^,^131^,^132^ Mechanistic studies have revealed that H_2_S mitigates reperfusion-associated oxidative damage through a triple synergistic effect: Firstly, by activating the biosynthetic pathway of antioxidant enzymes, such as catalase and SOD^133^; secondly, by promoting GSH secretion and its mitochondria-targeted transport^134^; and thirdly, by inhibiting the expression of NADPH oxidase and thus blocking the source of ROS generation.^135^

In the pathologic process of CIRI, the dynamic changes of MDA and SOD, as core biomarkers of OS, have important diagnostic value.^136^,^137^ Experimental studies have demonstrated that in the MCAO model, H_2_S exerts neuroprotective effects through a dual regulatory mechanism: On the one hand, by upregulating the expression level of GPx and modulating the balance of MDA metabolism, and on the other hand, by significantly augmenting the kinetic parameters of SOD enzyme activity, which can effectively dampen the neuronal damage mediated by excess ROS.^138^,^139^ At the molecular mechanistic level, MDA, as the terminal product of lipid peroxidation, inhibits mitochondrial respiratory chain activity and compromises mitochondrial membrane integrity, while SOD, functioning as the pivotal enzymatic component of antioxidant defense systems, exhibits attenuated catalytic activity that directly impairs cellular free radical scavenging capacity.^60^,^140^ Notably, brain tissues undergoing IRI showed characteristic changes: An increase in MDA content accompanied by a significant decrease in SOD activity.^141^,^142^,^143^ Further studies revealed that H_2_S not only acts by regulating the antioxidant enzyme system, but also directly participates in the free radical scavenging reaction. Geng’s team demonstrated through a cardiomyocyte model that NaHS was able to effectively inhibit isoproterenol-induced myocardial I/R by nucleophilic reactions with superoxide anion and hydrogen peroxide in the myocardial I/R model in the lipid peroxidation chain reaction, and its mechanism of action is closely related to the blockade of the MDA production pathway.^144^

Molecular mechanism studies have shown that Fu’s team^145^ systematically revealed the pathophysiological mechanism of endogenous H_2_S signaling axis in LIRI by establishing a rat lung I/R model. The results showed that exogenous H_2_S could enhance the resistance to LIRI in rats by directly or indirectly scavenging ROS and regulating endogenous antioxidant enzymes. Elevated MDA levels and decreased SOD activity facilitate lipid peroxidation and increase renal cell apoptosis by upregulating NF-κB, IL-2, and TLR4, thereby stimulating inflammatory responses.^146^ Animal experiments demonstrated that during kidney I/R, serum and tissue expression levels of ILs, TNF-α, MDA and other indicators of inflammation are markedly elevated, accompanied by decreased SOD or activity and tubular necrosis. However, H_2_S donor sodium sulfide (Na_2_S) treatment significantly reduced inflammation, OS, and kidney damage.^147^

In the investigation of H_2_S-mediated neuroprotective mechanisms against CIRI, scholars have discovered that its mechanism may be closely associated with the regulation of autophagy. The research team led by Zhu pioneeringly revealed the promotive effect of NaHS on autophagosome degradation by establishing both murine and cellular models of CIRI.^148^ Notably, co-administration of H_2_S with the autophagy-lysosomal inhibitor bafilomycin A1 demonstrated marked enhancement of autophagic flux concomitant with cerebral injury mitigation, in stark contrast to the aggravated pathological outcomes observed with bafilomycin A1 monotherapy. This pharmacological evidence indicates that dysregulated autophagic activation exacerbates CIRI, while H_2_S confers neuroprotection through modulation of autophagosome over-accumulation.^149^ Subsequent investigations have further corroborated that in the MCAO model, the elevated LC3-II/LC3-I ratio and reduced p62 protein expression serve as established biomarkers of autophagic activation.^150^,^151^,^152^ Of particular scientific interest, L-cysteine demonstrates significant upregulation of LC3-II and Beclin1 expression under ischemic/hypoxic conditions, concomitant with suppression of p62, ROS, MDA, and pro-inflammatory cytokines. These neuroprotective effects were abolished by autophagy inhibitors and showed a close correlation with endogenous H_2_S production, demonstrating that L-cysteine achieves neuroprotection through H_2_S-mediated activation of autophagic pathways.^153^ This cumulative research has not only delineated the molecular circuitry underlying H_2_S-mediated autophagy regulation, but also laid a mechanistic foundation for advancing therapeutic innovations that pharmacologically target autophagic flux to achieve neuroprotection.

### Dual biological role of hydrogen sulfide in ischemia–reperfusion injury

#### Concentration-dependent dual effects of neuroprotection and toxicity

Based on long-term research evidence, H_2_S has been validated as a novel neuromodulator and potential neuroprotective gasotransmitter with multifaceted biological effects across cell cultures, animal studies, and even human clinical observations. A study indicated that H_2_S exerts physiological regulatory effects within a broad concentration range (10–300 μM).^154^ Notably, in both *in vivo* and *in vitro* models of CIRI, H_2_S exhibits a concentration-dependent dual regulatory role. Current research consistently demonstrates that at low concentrations (e.g., physiological levels), H_2_S confers neuroprotection through multiple mechanisms, including but not limited to: Anti-apoptotic effects to reduce infarct volume, improved neurological outcomes, downregulation of OS and inflammatory cytokine expression, modulation of autophagy, and promotion of vasodilation and angiogenesis.^155^ However, at abnormally elevated concentrations (e.g., 180 μmol/kg via NaHS donor), H_2_S manifests neurotoxicity, significantly exacerbating neuronal damage.^156^ This “concentration window” effect underscores the necessity for precise dose control in clinical applications of H_2_S.

#### The dual regulatory role of hydrogen sulfide in autophagy

Autophagy acts as a “double-edged sword” for cells. Moderate autophagy facilitates the clearance of damaged organelles and promotes cell survival, whereas excessive autophagy may trigger cell death.^157^ Autophagy, as a highly regulated cellular process, is extensively involved in diverse pathophysiological mechanisms, with CIRI being one of its critical regulatory contexts.^158^,^159^ Regarding the neuroprotective mechanisms of exogenous H_2_S, existing evidence suggests its effects may be mediated through the modulation of autophagic activity. For instance, in the MCAO model, H_2_S has been shown to suppress overactivated autophagy, manifested by a decreased ratio of autophagy markers LC3-II/LC3-I and upregulated expression of the autophagy substrate p62 protein. However, in a spinal cord IRI model, NaHS (30 μmol/kg) pretreatment for 48 hours induced protective autophagy through downregulation of miR-30c and upregulation of Beclin-1 and LC3-II expression.^160^ This paradoxical phenomenon indicates that H_2_S-mediated autophagy regulation exhibits concentration dependence and tissue specificity, with its bidirectional regulatory properties potentially stemming from pathological differences between animal models or H_2_S dose threshold effects.

A study has further elucidated the intricate regulatory network of autophagy in MIRI. Utilizing an H9C2 rat cardiac myoblast model, Loos et al.^161^ demonstrated that mild ischemia simultaneously activates both autophagy and apoptosis pathways. Despite increased apoptotic and necrotic cell death under these conditions, upregulated autophagy still exhibited cardioprotective effects. In contrast, moderate to severe ischemia failed to effectively induce autophagy, suggesting a damage severity-dependent protective mechanism. Notably, the functional regulation of autophagy in the cardiac system displays significant age-related heterogeneity.^162^ For instance, Xiao et al.^163^ observed in neonatal cardiomyocytes that exogenous H_2_S suppresses autophagic activity by activating mTOR signaling, thereby attenuating IRI. Conversely, Chen’s team^164^ reported an opposing mechanism in aged cardiac models—H_2_S enhances autophagic flux via the AMPK/mTOR pathway to restore cardioprotective capacity. Based on these findings, a research team proposed a dynamic regulatory hypothesis: H_2_S acts as an “autophagic switch” during I/R processes. During the ischemic phase, when autophagy exerts adaptive protection, H_2_S maintains autophagic flux through AMPK activation; whereas during reperfusion, when autophagy transitions to a pathological mediator, H_2_S inhibits its hyperactivation via mTOR-related pathways to achieve precise radioprotective modulation.^165^

Previous studies have demonstrated that excessive activation of autophagy during HIRI can trigger autophagic cell death, and its effective suppression has been shown to significantly alleviate liver injury.^166^,^167^ However, it is critical to emphasize that moderate autophagy, as an endogenous protective mechanism, plays an essential role in maintaining hepatocyte homeostasis. Research indicates that H_2_S exerts hepatoprotective effects by inhibiting autophagic flux. Intriguingly, rapamycin enhances protective outcomes by counteracting H_2_S S-mediated autophagy inhibition, a paradoxical phenomenon suggesting that H_2_S acts through multi-layered regulatory mechanisms.^124^ Further investigations reveal a dose-dependent dual effect of H_2_S on autophagy modulation: Moderate inhibition confers cytoprotection, whereas excessive suppression may lead to adverse consequences. This dose-dependent divergence may be closely associated with pathological microenvironmental changes across different ischemic time windows. Notably, in various liver disease models, H_2_S exhibits significant heterogeneity in its regulatory direction toward autophagy—either promoting or suppressing it. Potential mechanisms underlying this phenomenon include: (1) dynamic fluctuations in local H_2_S concentrations; (2) differential baseline autophagy levels at distinct disease progression stages; and (3) variations in energy status and OS intensity of target cells during pathological processes.^168^ These findings underscore the necessity for clinical interventions to implement precise temporal regulation tailored to specific pathological phases.

In summary, H_2_S ameliorates IRI by dynamically regulating autophagic activity. However, its regulatory direction (promotion or inhibition) and effect intensity are governed by multiple factors, including tissue-specific basal autophagy levels, H_2_S concentration gradient effects, ischemia duration, and pathological heterogeneity of experimental models. These findings underscore that H_2_S-targeted therapies require precise modulation based on a “tissue-concentration-time-pathology” four-dimensional framework.

### Therapeutic potential of hydrogen sulfide in ischemic diseases

**Figure 4:**
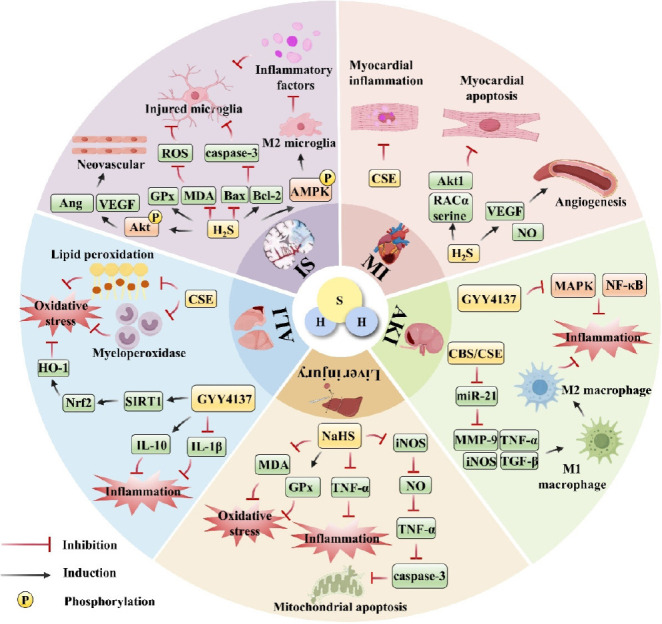
Therapeutic potential of H_2_S in ischemic diseases. H_2_S can exert protective effects in ischemic stroke (IS), myocardial infarction (MI), acute kidney injury (AKI), liver injury, and acute lung injury (ALI) by regulating different signaling pathways. Created with Microsoft PowerPoint 2021, some of the image materials are from Home for Researchers (www.home-for-researchers.com). Akt: Protein kinase B; AMPK: AMP-activated protein kinase; Ang: angiopoietin; Bax: Bcl-2 associated X protein; Bcl-2: B-cell lymphoma 2; CBS: cystathionine-β-synthase; CSE: cystathionine-γ-cleaving enzyme; GPx: glutathione peroxidase; H_2_S: hydrogen sulfide; HO-1: heme oxygenase-1; IL-10: interleukin 10; IL-1β: interleukin 1β; iNOS: inducible nitric oxide synthase; MAPK: mitogen-activated protein kinase; MDA: malondialdehyde; MMP-9: matrix metalloproteinase-9; NF-κB: nuclear factor κB; NO: nitric oxide; ROS: reactive oxygen species; SIRT1; sirtuin1; TGF-β: transforming growth factor β; TNF-α: tumor necrosis factor α; VEGF: vascular endothelial growth factor.

Building on these mechanistic insights into H_2_S in IRI, current evidence highlights its significant therapeutic promise for ischemic pathologies. This review provides a concise overview of H_2_S-mediated protection across multiple organ systems, spanning IS, myocardial infarction (MI), AKI, ALI and liver injury, highlighting its multi-organ protective efficacy (**Figure 4**).

#### Ischemic stroke

Building upon in-depth investigations into the neuroprotective mechanisms of H_2_S, this gasotransmitter has been identified as a pivotal regulator in IS pathology. Its core neuroprotective functions are manifested through two principal mechanisms: Primarily, H_2_S exerts fundamental protective effects by maintaining cerebrovascular tone homeostasis and endothelial barrier integrity. Clinical evidence demonstrates that decreased H_2_S levels significantly correlate with endothelial dysfunction, directly exacerbating IS progression.^169^ Secondly, preclinical studies have confirmed that exogenous H_2_S supplementation effectively promotes angiogenesis and neural network remodeling in murine models, highlighting its multifaceted cerebral protective capabilities.^170^

The biosynthesis of H_2_S predominantly relies on the catalytic activity of CBS within the CNS.^171^ Notably, in the classical MCAO model of stroke, CBS overexpression not only elevates cerebral H_2_S concentrations but also substantially mitigates ischemic injury through mechanisms involving suppression of microglial overactivation and downregulation of pro-inflammatory cytokine storms.^61^ Mechanistically, H_2_S alleviates CIRI in rats by specifically inhibiting the phosphorylation activation of dual sites (Thr436 and Ser575) on Rho-associated coiled-coil-containing protein kinase-2 kinase, thereby blocking downstream apoptotic signaling cascades.^172^ Of particular translational significance, total flavonoids from Rhododendron have been discovered to activate the CBS/H_2_S signaling axis, dynamically modulating the balance of A1/A2 astrocyte phenotypic transformation, thereby offering novel therapeutic targets for ischemic brain injury repair.^173^ A recent study revealed that zinc sulfide nanoparticles—zinc sulfide-polyethylene glycol (PEG) nanoparticles (ZnS-PEG NPs, H_2_S-controlled release donors) demonstrate significant neuroprotective mechanisms by markedly suppressing OS injury in oxygen glucose deprivation/reoxygenation models, effectively reducing neuronal apoptosis rates. Further investigation uncovered a dual regulatory effect of this nanoplatform on microglia: (1) By scavenging oxidative metabolites such as MDA and ROS, it alleviates cellular OS; (2) Through activation of the AMPK/NF-κB signaling pathway, it induces polarization of microglia toward the M2 anti-inflammatory phenotype, concurrently decreasing the secretion of pro-inflammatory cytokines including TNF-α and IL-6. Notably, this nanomaterial also exhibits pro-angiogenic properties. Mechanistic studies demonstrate that ZnS-PEG NPs significantly upregulate the expression of angiopoietin and vascular endothelial growth factor via activation of the Akt phosphorylation signaling pathway, thereby promoting endothelial cell proliferation and migration.^174^

Furthermore, H_2_S has demonstrated its therapeutic potential in mitigating early BBB damage and cerebral edema during focal cerebral ischemia, thereby exerting neuroprotective effects.^175^,^176^ Jiang et al.^175^ revealed that during CIRI, the endogenous H_2_S production system mediated by CSE and CAT/3-MST plays a critical regulatory role in maintaining early BBB integrity. H_2_S alleviates BBB damage in pathological conditions such as cerebral ischemia, subarachnoid hemorrhage, CNS infections, and cardiac arrest by inhibiting the degradation of tight junction proteins. Mechanistic studies indicate that H_2_S effectively reduces vasogenic cerebral edema through dual pathways: Suppressing the activation of matrix metalloproteinase-9 and downregulating abnormal expression of aquaporin-4. Notably, Cui’s team^177^ utilized GYY4137 to validate these findings, demonstrating that this compound stabilizes tight junction protein expression, thereby preserving BBB structural and functional integrity. These discoveries not only elucidate the molecular network underlying H_2_S-mediated cerebrovascular protection but also provide experimental evidence for developing targeted stroke therapies based on H_2_S signaling modulation.

#### Myocardial infarction

MI is caused by sustained myocardial hypoxia, ultimately leading to acute tissue ischemia and irreversible cardiomyocyte death. Recent studies indicate that exogenous supplementation of H_2_S or modulation of its endogenous production can significantly improve cardiac function, alleviate IRI, and reduce complications such as arrhythmias, heart failure, myocardial hypertrophy, and fibrosis.^4^ In an IRI mouse model, exogenous H_2_S treatment reduced myocardial infarct size and preserved left ventricular systolic function. Concurrently, cardiac-specific overexpression of CSE—a key enzyme for endogenous H_2_S synthesis—effectively suppressed myocardial inflammation, thereby protecting the structural and functional integrity of the myocardium and mitochondria.178 Further investigations revealed that the cardioprotective effects of H_2_S may involve activation of Ras-related C3 botulinum toxin substrate 1 α-serine/threonine-protein kinase and promotion of nuclear translocation of nuclear respiratory factor 1 and *Nrf2*. These mechanisms synergistically enhance antioxidant signaling pathway activity, inhibit apoptosis, and promote mitochondrial biosynthesis.^179^ Notably, a 12-week intervention with DATS (an H_2_S donor) not only improved left ventricular function and attenuated adverse remodeling but also promoted angiogenesis by enhancing the vascular endothelial growth factor-NO signaling pathway.^180^ These findings systematically demonstrate the critical role of endogenous H_2_S in maintaining fundamental cardiac physiological functions.

#### Acute kidney injury

RIRI constitutes a critical pathological basis for AKI, characterized by distinctive pathological alterations including intracellular calcium overload, ATP depletion, ROS burst, apoptotic cascade reactions, and cytokine storm.^181^ Studies demonstrate that endogenous H_2_S can partially counteract these injury processes through multiple pathways. Notably, under RIRI conditions, local H_2_S biosynthesis in kidneys is significantly impaired, while exogenous supplementation of H_2_S donors (e.g., NaHS, GYY4137) effectively ameliorates pathological progression.^34^,^181^,^182^ Experimental evidence confirms that NaHS exerts reno-protective effects through its anti-apoptotic, anti-inflammatory, and antioxidant properties. GYY4137 alleviates tissue inflammatory damage by suppressing excessive activation of the MAPK/NF-κB signaling pathway. The mitochondria-targeted donor AP39 significantly reduces renal tubular epithelial cell damage in IR models by specifically antagonizing glucose oxidase-induced ROS generation.^183^ These findings suggest that targeted delivery of H_2_S to specific subcellular structures may become crucial for optimizing therapeutic strategies.

In aged RIRI models, functional deficiencies in the endogenous H_2_S system are closely associated with abnormal miR-21 upregulation. This microRNA drives macrophage polarization towards pro-inflammatory M1 phenotype and promotes matrix metalloproteinase-9 expression, ultimately leading to renal interstitial fibrosis and vascular dysfunction. Exogenous H_2_S intervention significantly inhibits miR-21 overexpression, and blocks endothelial-mesenchymal transition, while improving renal microvascular density and hemodynamic function. Further mechanistic studies reveal that anti-miR-21 therapy using locked nucleic acid technology upregulates H_2_S synthase (CBS/CSE) expression *in vitro* and effectively suppresses pro-fibrotic factors (TNF-α, inducible NO synthase (iNOS), transforming growth factor β, and matrix metalloproteinase-9) *in vivo*. This approach promotes macrophage transition to reparative M2 phenotype and ultimately restores renal blood flow.^184^

#### Liver injury

HIRI represents a critical pathological mechanism contributing to severe liver damage and mortality in clinical settings. This injury plays a central role in the development of postoperative complications following liver resection and transplantation.^12^ Emerging evidence highlights the therapeutic potential of H_2_S in mitigating HIRI during transplantation. Specifically, H_2_S exerts protective effects by modulating key cellular and molecular pathways, including those associated with microcirculatory dysfunction, mitochondrial impairment, inflammatory cascades, cellular injury/death, and destructive signaling cascades. Concurrently, H_2_S activates cytoprotective mechanisms to counteract these pathological processes.^185^ A study has confirmed that the NaHS exerts significant protective effects on the liver.^186^ Serum biochemical tests demonstrated that this compound effectively reduces serum alanine aminotransferase levels in experimental animals, a finding further supported by morphological evidence from liver histopathological assessments. Mechanistic investigations revealed that the hepatoprotective effects of H_2_S involve multi-dimensional regulatory mechanisms: Notably alleviating OS through restoration of endogenous antioxidant enzyme activities such as GPx, while concurrently reducing oxidative damage markers including MDA; effectively suppressing the expression of pro-inflammatory factors such as TNF-α in inflammatory regulation; and significantly downregulating protein expression levels of iNOS and the key apoptotic executor caspase-3. These molecular-level regulatory mechanisms collectively constitute the hepatic protective network of H_2_S.

#### Acute lung injury

Emerging evidence extends the protective effects of H_2_S donors beyond hepatic transplantation to pulmonary graft models. During lung transplantation, donor lungs undergo cold ischemia and reperfusion after acquisition, a process that often leads to LIRI, which in turn leads to ALI and affects the function and survival of the transplanted lung. In a rat orthotopic lung transplantation study, recipient pretreatment with NaHS (14 μmol/kg) administered intraperitoneally 3 hours pre-graft implantation and 15 minutes pre-reperfusion significantly enhanced post-transplant pulmonary function and tissue preservation compared to untreated controls. Mechanistically, these improvements correlated with elevated graft tissue H_2_S levels and CSE protein expression, accompanied by reduced lipid peroxidation, suppressed myeloperoxidase activity, and a favorable cytokine shift marked by decreased IL-1β and increased IL-10 production.^187^ In a complementary experiment, pharmacological inhibition of endogenous H_2_S synthesis via pre-transplant administration of propargylglycine (37.5 mg/kg, a selective CSE inhibitor) exacerbated cold IRI, manifesting as aggravated pulmonary dysfunction and histological damage.^187^ These findings collectively demonstrate that activation of the CSE/H_2_S axis in transplant recipients confers protection against cold IRI through mechanisms involving redox homeostasis regulation and inflammatory response modulation.

Additionally, in the diabetic lung I/R model, the rats treated with GYY4137 showed improved lung function recovery, reduced oxidative damage, inflammation, and cell apoptosis. Mechanistic studies demonstrated that GYY4137 activated the SIRT1 signaling pathway, which subsequently upregulated the antioxidant signaling pathways mediated by *Nrf2*/HO-1 and eNOS, thereby reducing cell apoptosis and inflammation, and ultimately protecting lung function under diabetic IRI conditions.^102^

## Development of Hydrogen Sulfide Drugs

Accumulating research evidence indicates that H_2_S at physiological concentrations exhibits unique cytoprotective properties in biological systems through multidimensional physiological mechanisms, including regulation of apoptotic pathways, precise modulation of autophagy, effective suppression of OS responses, and regulation of inflammatory signaling pathways. With a deepening understanding of its core biological functions (vasodilation, cytoprotection, antioxidant and anti-inflammatory effects) and related signaling mechanisms, this endogenous gasotransmitter demonstrates broad clinical application potential. Current research focuses on the rational design of novel H_2_S donor molecules to achieve therapeutic goals of precise delivery and controllable release.

In donor development, researchers aim to construct H_2_S prodrug systems with sustained-release characteristics. Studies confirm that donor systems featuring stable sulfur-storage structures and sustained H_2_S release better meet clinical needs. Several administration strategies have been established: (1) Thiol-activated donors: Represented by GYY4137, these compounds gradually release H_2_S through endogenous thiol (e.g., GSH)-mediated enzymatic reactions, demonstrating spatiotemporally controlled release characteristics^188^; (2) Polysulfide systems: Organic polysulfides such as DATS generate H_2_S through metabolic conversion *in vivo*, effectively prolonging drug duration,^189^,^190^,^191^ with oral or intravenous administration enabling systemic H_2_S delivery; (3) Stimuli-responsive smart donors: Recently developed synthetic organic molecules can detect specific microenvironmental stimuli to trigger selective H_2_S release. Additionally, nanotechnology has been widely applied in H_2_S donor development. These molecular design breakthroughs significantly enhance the targeting and safety of H_2_S therapy, laying the foundation for clinical translation of this therapeutic strategy.

## Pharmacological Effects of Hydrogen Sulfide in Ischemia–Reperfusion Injury

Building upon groundbreaking discoveries regarding the unique therapeutic value of H_2_S as a critical gasotransmitter in disease treatment–including its regulatory roles in redox balance, mitochondrial function preservation, and inflammatory microenvironment remodeling–significant progress in recent years has spurred the innovative design of targeted small-molecule organosulfur compounds and intelligent H_2_S delivery systems (**Table 1**). These technological breakthroughs have not only deepened our understanding of the spatiotemporal specificity within H_2_S signaling networks, but also established a molecular foundation for developing next-generation therapeutic strategies based on precise gas molecule modulation.

**Table 1:** Pharmacological effects of H_2_S in ischemia–reperfusion injury

Intervention	Regulation	Effect or function	Reference
HSDF-NH_2_	TNF-α↓, IL-1β↓, ROS↓, glial fiber acidic protein↓	Penetrating the BBB, reducing infarct volume, decreasing apoptosis and attenuating OS	84
PSA@ADT-OH	ROS↓, IL-6↓, TNF-α↓	Inhibiting OS and inflammation	202
A6	Cell viability↑	Reducing the area of cerebral infarction	203
NaHS	H_2_S↑, GDNF↑, BDNF↑, Promoting astrocyte transformation toward the A2 phenotype JNK phosphorylation↓, CSE/eNOS↑, iNOS↓, Akt↑, MDA↓, GPx↑	Protecting the integrity of the BBB	204
		Neuroprotection	206
		Neuroprotection	208
		Anti-apoptosis	209
		Improving cardiac contractile function	210
		Alleviating OS and protecting mitochondrial function	211
		Restoring the oxidative-antioxidative imbalance	214
STS	Caspase-3/9↓, PI3K/mTOR/KATP↑, PI3K/AKT/GSK33↑, pro-apoptotic factors↓, inflammatory mediators↓, CD68^+^↓, MPO^+^↓, mPTP↓, ROS↓	Neuroprotection	217
		Reducing myocardial infarct size	219
		Preserving mitochondrial functional integrity	220
		Anti-apoptosis and anti-inflammation	221
		Preventing mitochondrial-related apoptosis and reducing OS	223
SAC	ERK1/2↓, JNK/p38↓, Nrf2/ARE↑, HO-1↑, GPx↑, SOD↑, CAT↑, iNOS↓, GFAP↓	Neuroprotection and anti-oxidative stress	224
		Anti-inflammation	225
SPRC	CD24/Src/Fak/Pyk2↑, Caspase-12↓, GRP78↓, CHOP↓, Akt↑, SOD↑, ROS↓, PI3K/Akt↑, MEK-ERK↑	Anti-inflammation	227
MTC		Anti-apoptosis and anti-oxidative stress	232
		Promoting proliferation of neural stem cells	138
AP39	H_2_S↑, DNF-TrkB↑, NGF-TrkB↑, proNGF-p75NTR-sortilin↓, GFAP↓, Bcl-10↓, DDIT3↓	Mitochondrial protective	233
		Neuroprotection, anti-apoptosis and anti-inflammation	234
		Anti-apoptosis	236
GYY4137	p38 MAPK↓, ERK1/2↓, JNK↓, Bax↓, Bcl-2↑, caspase-3↓	Anti-apoptosis	107

ADT-OH: 5-(4-Hydroxyphenyl)-3H-1,2-dithiocyclopentene-3-thiolide; Akt: protein kinase B; ARE: antioxidant response element; Bax: Bcl-2 associated X protein; BBB: blood-brain barrier; Bcl-10: B-cell lymphoma 10; Bcl-2: B-cell lymphoma 2; BDNF: brain-derived neurotrophic factor; CAT: cysteine aminotransferase; CHOP: homologous protein; CSE: cystathionine gamma-lyase; DDIT3: DNA damage inducible transcript 3; eNOS: endothelial nitric oxide synthase; ERK: extracellular signal-regulated protein kinase; ERK1/2: extracellular signal-regulated protein kinase 1/2; GDNF: glial-derived neurotrophic factor; GFAP: glial fibrillary acidic protein; GPx: glutathione peroxidase; GRP78: glucose-regulated protein 78; GSK33: glycogen synthase kinase 3β; GYY4137: morpholin-4-ium 4-methoxyphenyl (morpholino) phosphinodithioate; H_2_S: hydrogen sulfide; HO-1: hemoglobin oxygenase-1; IL-1β: interleukin 1β; IL-6: interleukin 6; iNOS: inducible nitric oxide synthase; JNK: c-Jun N-terminal kinase; KATP: ATP-sensitive potassium channel; MDA: malondialdehyde; MEK: mitogen-activated protein kinase; MPO: myeloperoxidase; MTC: S-(4-fluorobenzyl)-N-(3:4:5-trimethoxybenzoyl)-L-cysteine; mTOR: mammalian target of rapamycin; NaHS: sodium hydrosulfide; Nrf2: nuclear factor erythroid 2-related factor 2; NG F: nerve growth factor; OS: oxidative stress; PI3K: phosphatidylinositol 3-kinase; p75NTR: p75 neurotrophin receptor; ROS: reactive oxygen species; SAC: S-allyl cysteine; SOD: superoxide dismutase; SPRC: S-propargyl cysteine; STS: sodium thiosulfate; TNF-α: tumor necrosis factor α.

### Hydrogen sulfide donors

#### HSDF-NH_2_

Although the therapeutic potential of H_2_S in IRI has been well-documented, its clinical application faces substantial challenges due to inherent physicochemical limitations. The physicochemical properties of H_2_S, characterized by high volatility and hyperreactivity, create substantial technical barriers in achieving precise spatiotemporal control over its concentration and dosage. This critical limitation significantly impedes both clinical translation of H_2_S-based therapies and comprehensive elucidation of its underlying molecular mechanisms. This has seriously hindered the clinical use of H_2_S and its pharmacological mechanism. The HSD-B donor platform engineered by Zhang et al.^192^ pioneered real-time visualization and dynamic modulation of exogenous H_2_S liberation within controlled *in vitro* environments. Based on this technological breakthrough, the team successfully constructed a novel H_2_S donor HSDF-NH_2_ by introducing hydrophilic amino-substituted triphenylphosphine groups. This system realized the real-time tracking and precise quantification of the H_2_S release process, which provided an innovative solution to the targeting delivery challenge. Animal experiments showed that HSDF-NH_2_ has superior BBB penetration ability, and its accumulation in brain tissues showed a time-dependent increase. Mechanistic studies demonstrated that the donor significantly inhibited the levels of pro-inflammatory cytokines (TNF-α, IL-1β) and ROS, while concurrently reducing the expression of glial fibrillary acidic protein (GFAP). Pathological analysis confirmed that the HSDF-NH_2_ treatment group exhibited a decreased number of apoptotic cells and reduced infarct volume in brain tissue, demonstrating significant neuroprotective effects. Of particular note, HSDF-NH_2_ demonstrated excellent biocompatibility, providing a critical safety rationale for its clinical translation.^193^

#### PSA@ADT-OH

5-(4-Hydroxyphenyl)-3H-1,2-dithiocyclopentene-3-thiolide (ADT-OH), a prototypical sustained-release H_2_S donor.^194^ Recent studies have demonstrated that the combined application of ADT-OH with tissue plasminogen activator produces synergistic effects. Animal experimental results revealed that co-administration of ADT-OH effectively mitigates tissue plasminogen activator-induced disruption of BBB integrity while significantly improving neurological functional recovery in MCAO model mice following tissue plasminogen activator thrombolytic therapy.^195^ In addition to drug thrombolytic therapy, photothermal therapy (PTT) as a method for treating thrombosis has received extensive attention due to its spatiotemporal selectivity and minimally invasive nature.^196^,^197^ However, the high temperature generated by PTT may exacerbate vascular inflammation, hinder the vascular repair mechanism, and increase the risk of thrombus recurrence after PTT.^198^,^199^,^200^ H_2_S can inhibit platelet adhesion to collagen or fibrin, exerting an anti-thrombotic effect,^201^ and can work synergistically with PTT to achieve more effective thrombolytic treatment. A research team successfully developed a polymer-based carrier system (PSA) utilizing perylene diimide as the structural framework, which effectively encapsulated the H_2_S donor ADT-OH to form a nanocomposite drug designated as PSA@ADT-OH.^202^ By establishing a dynamic thrombus model simulating pathological high-shear stress conditions, experimental results demonstrated that P-selectin-targeting ligand-modified PSA carriers could overcome fluid shear stress interference and achieve selective aggregation at thrombus formation sites. Furthermore, validation in a human umbilical vein endothelial cell model confirmed that this delivery system enabled spatiotemporal precision in drug localization within thrombotic microenvironments through dynamic ligand-receptor interactions. The study further validated the interventional efficacy of PSA@ADT-OH and ADT-OH by constructing an *in vitro* thromboinflammatory microenvironment model with elevated ROS concentrations. Fluorescence quantification revealed significant attenuation of fluorescence signal intensity post PSA@ADT-OH/ADT-OH treatment, indicating H_2_S derived from these compounds effectively scavenges intracellular ROS and mitigates OS damage. Concurrently, marked reductions in pro-inflammatory cytokines IL-6 and TNF-α were observed, demonstrating H_2_S-mediated suppression of the inflammatory cascade via redox homeostasis regulation.

In another study, a novel series of H_2_S-releasing nicotinic acid derivatives was successfully constructed by using nicotinic acid as the parent nucleus combined with the ADT-OH. This series of compounds exhibited significant neuroprotective activity in a glutamate-induced HT22 hippocampal neuronal injury model without showing cytotoxicity. Among them, the lead compound A6 demonstrated a dual advantage in the permanent MCAO model: Not only significantly enhancing neuronal survival, but also shrinking cerebral infarct volume, highlighting its clinical translational potential as a novel cerebral ischemic neuroprotective agent.^203^

#### Sodium hydrosulfide

As an important H_2_S donor, NaHS exerts multi-system protective effects in organisms through rapid release of H_2_S. Experimental studies have shown that this compound not only significantly elevated the H_2_S concentration in the ischemic brain region in the MCAO model, but also confirmed its protective effect on the BBB.^204^ Emerging mechanistic insights reveal that astrocytic populations exert dual regulatory control over CNS pathophysiology during IS progression.^205^ Mechanistically, neuroinflammatory microenvironments induce polarization of A1-type reactive astrocytes that exacerbate neuronal damage through pro-inflammatory cytokine release, whereas ischemic conditions promote an A2-dominant phenotype characterized by upregulated expression of glial-derived neurotrophic factor and brain-derived neurotrophic factor to confer neuroprotection.^206^ This dynamic equilibrium between astrocytic phenotypic switching operates as a critical regulatory nexus governing post-stroke neuronal survival and tissue repair. Emerging pharmacological evidence demonstrates that NaHS, acting as an exogenous H_2_S donor, facilitates astrocytic phenotypic switching from neurotoxic A1 to neuroprotective A2 polarization states. This mechanism was experimentally validated in Ding’s study^207^ using a cerebral I/R model. Through systematic quantification of GFAP (pan-astrocyte marker), complement component C3 (A1-specific indicator), and S100 calcium-binding protein A10 (S100a10, A2-associated biomarker), it was found that NaHS treatment significantly prevented the reduction of GFAP and S100a10 expression. By constructing a CSE gene conditional knocked out animal model, Li et al.^208^ revealed the crucial role of endogenous H_2_S signaling in regulating astrocyte phenotypic switching following CIRI. Through experiments using an LPS-induced neuroinflammation model combined with MCAO technology, it was found that CSE-knocked out mice exhibited increased GFAP cell density in the hippocampal region and elevated complement C3 protein levels compared to wild-type mice, confirming that endogenous H_2_S deficiency exacerbates activation of A1-type reactive astrocytes. In contrast, the NaHS treatment group displayed opposing regulatory effects: Reduced GFAP and C3 expression, alongside significant upregulation of S100a10 expression levels in both CSE-knocked out and wild-type groups. These findings demonstrate that endogenous H_2_S suppresses reactive astrocyte proliferation post-CIRI and promotes astrocyte transformation toward the A2 phenotype, thus exerting neuroprotective effects.

In the field of myocardial protection, NaHS demonstrated a triple defense mechanism: Inhibiting JNK2 phosphorylation and blocking the pro-apoptotic signaling cascade response^209^; improving cardiac contractile function by modulating the NO synthase system (CSE/eNOS upregulation and iNOS downregulation)^210^; and activating the Akt pathway to alleviate OS and protect mitochondrial function, which significantly promotes the recovery of cardiac function.^211^ Notably, pretreatment with NaHS resulted in a reduction of myocardial infarct size.^212^ While the synergistic effect of the dual antioxidant/anti-inflammatory mechanism resulted in a reduction of the reperfusion injury index.^57^,^104^ Emerging research demonstrates that NaHS (100 μM) as an H_2_S donor enhances cardiomyocyte viability, total antioxidant capacity, and tetrahydrobiopterin levels while reducing ROS production, MDA content, 8-hydroxy-2’-deoxyguanosine, and dihydrobiopterin concentrations in cardiomyocytes exposed to hyperglycemia, hypoxia-reoxygenation, or their combination. Mechanistically, H_2_S donor treatment upregulates both expression and activity of peroxisome proliferator-activated receptor α, counteracts the downregulation of PPAR-γ, peroxisome proliferator-activated receptor-γ coactivator-1-α, AMPK, glucose transporter type 4, *Nrf2*, phosphorylated eNOS, SOD, and CAT, and suppresses Keap1, p47phox, and NADPH oxidase 4 expression. These coordinated effects mitigate OS markers and enhance antioxidant defenses, ultimately preserving cardiomyocyte ultrastructural integrity and protecting against hyperglycemia, hypoxia-reoxygenation, or dual insult-induced cellular damage.^213^

Furthermore, investigations demonstrated that the NaHS exhibits multi-organ protective effects in a renal I/R model. Experimental data revealed that NaHS effectively restored the oxidative-antioxidative imbalance by significantly reducing MDA levels and enhancing GPx activity. Pathological evaluations showed reduced apoptotic neurons in the hippocampal region, decreased cerebral tissue edema, attenuated cardiomyocyte necrosis, and diminished inflammatory infiltration. Pulmonary histopathological analysis indicated alleviated alveolar collapse and reduced neutrophil infiltration.^214^ These systematic findings elucidate the multi-target regulatory properties of the H_2_S signaling system in organ protection.

#### Sodium thiosulfate

Sodium thiosulfate (STS), the primary oxidative metabolite of H_2_S, has been widely applied in cancer treatment and urolithiasis management; accumulating evidence now indicates that STS possesses antioxidant and anti-inflammatory properties, positioning it as a potential therapeutic target for IRI clinical interventions.^215^ Research conducted by Marutani’s team in a CIRI mouse model revealed that STS exhibits high binding affinity with caspase-3 protein. Through a peroxynitrite-mediated persulfide exchange reaction at caspase-3’s active site, STS effectively inhibits the enzymatic activity of this protease, thereby conferring neuroprotective effects and demonstrating therapeutic potential for IRI clinical management.^216^ A study based on a carotid artery occlusion animal model systematically investigated the protective mechanisms of STS in CIRI associated with vascular calcification.^217^ Experimental results demonstrated that the STS intervention group showed a significant increase in cortical neuronal survival rate compared to the control group, along with reduced leakage of LDH and creatine kinase in ischemic brain regions. Molecular mechanism studies revealed suppressed activities of caspase-3/9 and neuroprotective effects comparable to mechanical preconditioning in *ex vivo* brain slice models. Notably, STS effectively alleviated IRI in both adenine-induced metabolic calcification models and high-fat diet-induced arterial calcification models.

As a H_2_S donor with anti-inflammatory properties, STS enhances endogenous H_2_S bioavailability.^218^ Mechanistic investigations further revealed that STS confers myocardial protection against MIRI by activating the PI3K/mTOR/ATP-sensitive potassium channel signaling axis, which mediates a significant reduction in myocardial infarct size.^219^ A recent study revealed that continuous STS administration in particulate matter less than 2.5 (PM_2.5_)-exposed cardiac I/R models ameliorated cardiac function in rats, accompanied by upregulated expression of the master regulatory gene peroxisome proliferator-activated receptor gamma coactivator 1α and enhanced mitochondrial mass. Furthermore, STS restored bioenergetic function and balanced mitochondrial fission-fusion dynamics. The metal-chelating capacity of STS further validated its therapeutic benefits by mitigating heavy metal deposition in mitochondria, thereby reducing OS and inflammation. Significantly, STS facilitated the clearance of damaged mitochondria through mitophagy activation. Mechanistically, STS conferred cardioprotection against IRI in PM_2.5_-exposed hearts by activating the PI3K/AKT/GSK-3β signaling pathway, which preserved mitochondrial functional integrity.^220^

A Recent study suggests that treatment with H_2_S donors in rat renal transplantation models may effectively improve donor kidney quality.^221^ Research based on STS-supplemented University of Wisconsin preservation solution demonstrates that this protocol operates through dual regulatory mechanisms: It not only significantly downregulates the expression of pro-apoptotic factors and inflammatory mediators in transplanted kidney tissues, but also enhances anti-apoptotic gene activity while suppressing macrophage (cluster of differentiation (CD)68^+^) and myeloperoxidase (MPO^+^) infiltration. These combined effects ultimately reduce cold IRI severity and promote post-transplant renal functional recovery. Notably, STS pretreatment may exert protection through mechanisms potentially involving inhibition of programmed cell death, mitigation of acute tubular necrosis, and regulation of neutrophil-mediated inflammatory responses. Experimental evidence indicates these protective effects correlate with alleviated OS and suppression of ferroptosis pathways, though precise molecular mechanisms require further validation via gain- and loss-of-function experiments. These findings offer novel strategic directions for optimizing organ preservation protocols, yet their clinical applicability awaits confirmation through translational research.^222^

STS exhibits protective functions in HIRI. Mechanistic studies reveal that STS maintains sustained H_2_S levels through controlled release modulated by hepatic tissue presence, effectively preventing donor-derived H_2_S oversaturation–a regulatory mechanism underlying its favorable safety profile. Furthermore, STS-released H_2_S specifically inhibits mitochondrial permeability transition pore opening during HIRI, thereby preventing mitochondrial-related apoptosis. The STS-mediated ROS scavenging mechanism achieves dual protective effects by substantially reducing lipid peroxidation and attenuating mitochondrial ultrastructural damage, which collectively preserves hepatic histoarchitecture at both molecular and pathological levels. Significantly, STS concurrently augments hepatic antioxidant reserves to counteract IRI-induced oxidative insults. This comprehensive antioxidant strategy synergistically preserves mitochondrial membrane integrity while mitigating parenchymal damage characteristic of HIRI.^223^

#### S-allyl cysteine

S-allylcysteine (SAC), recognized as the most bioactive organosulfur compound in aged garlic, exhibits antioxidant, anti-inflammatory, and neuroprotective properties, demonstrating significant therapeutic potential for intervention in cerebral ischemic disorders.^224^ In cerebral ischemia models, pre-treatment with SAC demonstrates multidimensional neuroprotective effects. This intervention strategy significantly reduces neuronal damage in the hippocampal CA1 region while simultaneously alleviating cerebral edema and reducing infarct size, with its mechanism potentially associated with inhibiting ROS burst. Pharmacological studies reveal that SAC administration prior to ischemia not only improves neurobehavioral scores but also achieves neuroprotection through inhibition of abnormal activation in the MAPK family signaling pathways, including ERK1/2 and the JNK/p38 pathways. At the level of the antioxidant defense system, SAC exhibits dual regulatory mechanisms: Activating the *Nrf2*/ARE signaling axis to significantly upregulate enzymes involved in GSH biosynthesis, including glutamate-cysteine ligase catalytic subunit and regulatory subunit, while inducing HO-1 expression; Enhancing the activity of various antioxidant enzyme systems, including GSH reductase, GPx, SOD, and CAT. Notably, this compound also effectively suppresses the expression of glial activation marker GFAP and pro-inflammatory mediator iNOS.^225^

In the study of cardiac IRI, experimental results revealed that SAC intervention induced a significant reduction in LDH release at 1 and 2 minutes post-reperfusion in the model group. Although no statistically significant differences were observed between groups at 5 and 10 minutes, this time-dependent alteration suggests that SAC modulates the injury mechanism primarily by delaying enzymatic release processes rather than completely inhibiting them. Notably, the marked decrease in total LDH release confirmed SAC’s protective effect in enhancing myocardial cells’ stress resistance, potentially through inducing endogenous antioxidants to counteract free radical-mediated damage. While its complete molecular targets remain unelucidated, these findings collectively demonstrate that SAC exerts cardioprotective effects via multi-target regulatory mechanisms.^226^

#### S-propargyl-cysteine

A study elucidates the neuroprotective mechanisms of S-propargyl-cysteine (SPRC), a novel H_2_S homeostasis regulator, in IS through its modulation of CD24-related signaling pathways. As a SAC derivative, SPRC demonstrates unique neuroinflammation-regulating properties. Experimental findings reveal that CD24 overexpression in BV2 microglial cell inflammatory models exerts dual-pathway regulation on inflammatory responses: (1) suppression of the NF-κB classical inflammatory pathway (evidenced by reduced IκBα phosphorylation levels), and (2) activation of Src/Fak/Pyk2 cascade signaling to enhance chemotactic migration of M2-polarized microglia. Mechanistic investigations indicate that SPRC specifically upregulates CD24 expression by enhancing the activity of CBS, a key endogenous H_2_S-synthesizing enzyme, thereby forming a multi-target regulatory network. This dual-channel regulatory mechanism not only inhibits pro-inflammatory factor release but also establishes a sustained anti-inflammatory microenvironment through enhanced chemotaxis of M2-type cells to lesion sites. These findings not only confirm CD24 as a novel therapeutic target for IS but also clarify SPRC’s coordinated action mechanism involving the synergistic interplay between the CD24/IκBα/NF-κB anti-inflammatory pathway and the CD24/Src/Fak/Pyk2 chemotaxis signaling axis, offering innovative strategies for cerebrovascular disease treatment.^227^ Similarly, SPRC has a protective effect on liver tissue in methionine/choline-deficient diet-induced nonalcoholic steatohepatitis mice, which is associated with the activation of the PI3K/Akt/*Nrf2*/HO-1 signaling pathway.^12^ In the LPS-induced neuroinflammatory model, SPRC effectively ameliorates spatial learning and memory impairments in experimental animals. This neuroprotective effect is closely associated with its ability to maintain the metabolic homeostasis of endogenous H_2_S.^228^,^229^ This distinctive dual mechanism of action confers significant translational potential, positioning it as a pivotal candidate for advancing multi-target therapeutic strategies against neuroinflammatory disorders.

#### S-(4-chlorobenzyl)-N-(3,4,5-trimethoxybenzoyl)-L-cysteine

MTC is a novel complex consisting of SAC and the natural polyphenolic compound gallic acid, which may produce neuroprotective effects against cerebral ischemic injury through a multi-target mechanism. Studies have demonstrated that gallic acid, serving as the core constituent of the complex, may play a pivotal role in neuroprotective processes owing to its distinctive antioxidant, anti-inflammatory, and antimicrobial properties. Notably, this naturally derived phenolic compound demonstrates high biosafety, thereby establishing a theoretical foundation for its therapeutic application in CNS disorders.^230^,^231^ Endoplasmic reticulum stress following IRI induces neuronal death, manifested by elevated levels of C/EBP homologous protein and glucose-regulated protein 78.^232^ Mechanistic studies have shown that MTC exerts neuroprotective effects through the synergistic action of multiple pathways. At the molecular level, the complex significantly down-regulated the expression of endoplasmic reticulum stress-related proteins (caspase-12, glucose-regulated protein 78, and C/EBP homologous protein), and enhanced Akt phosphorylation, effectively inhibiting I/R-induced programmed cell death. Its antioxidant properties were reflected in the PC12 cell model, which was able to enhance SOD activity and reduce the concentration of free radicals such as ROS. Animal experiments confirmed that after MCAO model rats received MTC intervention, the dual signaling pathways of PI3K/Akt and MEK-ERK were specifically activated, which was manifested by the increased number of axonal neonates and prolongation of axon length, and ultimately led to the increase of neuronal survival after CIRI by inhibiting apoptosis in the mitochondrial pathway, and promoting proliferation of neural stem cells, among other mechanisms.^138^ Based on the experimental results, MTC exhibited a significant therapeutic effect of inhibiting the inflammatory response in cerebral ischemia model rats, but its specific mechanism of action still requires in-depth pharmacological studies. Notably, the compound demonstrated both a favorable safety profile and reliable therapeutic potential, especially a superior therapeutic window compared with existing neuroprotective agents, which provides an important experimental basis and translational prospect for its development as a novel IS drug candidate.

#### AP39

AP39 is a mitochondria-targeted H_2_S donor that specifically releases H_2_S within mitochondria, thereby exerting mitochondrial protective effects.^233^ In a rat MCAO model, Pomierny et al.^234^ demonstrated that AP39 reduced infarct size, minimized neurological deficits and decreased the expression of microglia markers, and exhibited anti-inflammatory activity. In addition, AP39 enhanced the neurotrophic brain-derived neurotrophic factor-TrkB and nerve growth factor-TrkB pro-survival pathways and decreased the activity of the pro-apoptotic pro-nerve growth factor-p75 neurotrophin receptor-sortilin pathway. A recent study elucidating the relationship between IS and trace elements/minerals revealed that AP39 reduces potassium concentrations in post-stroke ischemic regions, yet paradoxically elevates potassium levels in normal tissues. This dual effect may stem from AP39’s modulation of potassium channels (e.g., potassium two pore domain channel subfamily K member 5), which are upregulated following ischemic injury.^235^ Collectively, these findings indicate that AP39 functions as a slow-release H_2_S donor, exerting antioxidant, anti-inflammatory, and anti-apoptotic neuroprotective effects through various targets.

Research in kidney transplantation has revealed the potential application value of AP39 in organ preservation and functional repair. The research team employed a porcine kidney xenotransplantation model and innovatively added AP39 to the preservation of blood during 21°C subnormothermic machine perfusion, systematically evaluating its impact on transplanted kidney function. Compared to the traditional model of 4-hour *ex vivo* hypothermic preservation followed by 4-hour normothermic reperfusion, subnormothermic perfusion with AP39-supplemented blood improved urine output, reduced tissue damage, and induced distinct pro-survival gene expression in porcine kidneys. For the first time, the study demonstrated that AP39 may exert its anti-apoptotic effects by downregulating the pro-apoptotic regulatory gene Bcl-10 and the transcriptional regulator of endoplasmic reticulum stress-induced apoptosis, DNA damage inducible transcript 3.^236^ Furthermore, AP39 supplementation enhanced hepatic viability during static cold storage. Following preservation, livers underwent 6-hour acellular normothermic machine perfusion, serving as a transplant simulation. AP39-treated livers exhibited reduced vascular resistance, lower levels of cellular injury (alanine aminotransferase/aspartate aminotransferase), and diminished apoptosis during this simulated transplantation. Additionally, AP39 administration promoted bile production, glucose metabolism, and energy charge (ATP content).^237^

#### GYY4137

GYY4137, a dual sulfur-containing compound functioning as a slow-releasing H_2_S donor, generates biological H_2_S through controlled hydrolysis. It has demonstrated cytoprotective effects across preclinical models of IRI, atherosclerosis, and neurodegenerative disorders through three principal mechanisms: Modulating OS, preserving organ function, and inhibiting apoptotic pathways.^238^ A study investigated the neuroprotective mechanism of H_2_S donor GYY4137 in focal CIRI. By establishing a rat MCAO model, it was found that the GYY4137 treatment group showed significant changes compared to the control group: The volume of cerebral infarction was reduced, the degree of brain edema was decreased, and the neurological function score improved. Mechanism studies revealed that GYY4137 exerted protective effects through three pathways: First, it significantly inhibits the key kinase p38 in the MAPK signaling pathway (evidenced by reduced phosphorylation levels), and suppresses the activation of ERK1/2 and JNK. Second, it modulates the balance of Bcl-2 family proteins by downregulating the expression of the pro-apoptotic protein Bax and upregulating the expression of the anti-apoptotic protein Bcl-2. Finally, it effectively suppresses the activity of caspase-3. These findings demonstrated that GYY4137 significantly alleviated neuronal programmed cell death caused by I/R by coordinating the regulation of MAPK signal transduction and mitochondrial-dependent apoptotic pathways.^107^

GYY4137 has demonstrated multifaceted therapeutic efficacy, with particular prominence in cardiovascular therapeutics where its cardioprotective effects against pathological processes such as MIRI and chronic heart failure have been substantiated through multiple preclinical investigations.^239^ Experimental evidence demonstrates that the GYY4137 exhibits cardioprotective properties independent of NO-mediated mechanisms. When administered at the end of the ischemic phase, this compound effectively reduces MI area to an extent statistically equivalent to Na_2_S treatment. Mechanistic investigations further revealed that in combination therapy, Na_2_S requires NO signaling pathway activation to exert its anti-infarction effects. Notably, the combined application of GYY4137 and Na_2_S generates synergistic cardioprotection surpassing monotherapy outcomes. This phenomenon not only confirms the existence of two distinct H_2_S-associated cardioprotective pathways but also provides the first evidence of their additive interaction through coordinated mechanisms. These findings offer crucial molecular insights and potential translational applications for optimizing H_2_S-based therapeutic strategies in cardiac protection.^240^

### Future applications of hydrogen sulfide

According to current research, the medical applications of H_2_S primarily focus on two approaches: endogenous regulation and exogenous supplementation. In clinical practice, the main administration methods involve direct inhalation of gaseous H_2_S and donor compounds. However, it should be noted that the inhalation approach presents significant limitations: H_2_S not only exhibits neurotoxicity but also possesses a characteristic rotten-egg odor that severely compromises patient compliance. More critically, precise control of alveolar inhalation concentration remains challenging. While donor compounds partially address safety concerns, they encounter obstacles in precise delivery due to complex pharmacokinetics and poor tissue targeting.^241^ Current clinical practice predominantly employs inorganic sulfides as H_2_S donors, yet their therapeutic utility is constrained by pharmacodynamic limitations including dose-dependent targeting inefficiency and potential systemic toxicity. While substantial progress has been made in developing small-molecule H_2_S donors, these compounds continue to demonstrate critical functional deficiencies, particularly in target specificity, pharmacokinetic half-life, chemical stability, and aqueous solubility under physiological conditions.^242^ This emerging paradigm has propelled the development of advanced delivery systems as a strategic focus for unlocking H_2_S’s therapeutic potential. Contemporary H_2_S-release platforms typically integrate donor molecules with biocompatible polymeric carriers through two principal engineering approaches: Covalent conjugation strategies and physical encapsulation techniques, employing sophisticated delivery vehicles such as liposomes,^243^ nanoparticles, and hydrogel matrices^244^ for optimized biological interfacing.

As an engineered solution for H_2_S delivery, nanocarriers demonstrate multifunctional advantages including enhanced pharmaceutical stability, optimized pharmacokinetic profiles, and spatiotemporal-controlled release capabilities. Among various nanotechnology platforms, polymeric nanoparticles fabricated from biodegradable copolymers–particularly poly (lactic-co-glycolic acid) and PEG derivatives–have emerged as particularly promising candidates due to their tunable drug release kinetics and exceptional biocompatibility.^245^,^246^ Nanoparticulate systems demonstrate precise modulation of H_2_S release kinetics through advanced nano-engineering approaches, particularly via physical encapsulation of H_2_S prodrugs. This nanotechnology-enabled strategy achieves dual optimization of metabolic clearance patterns and bioavailability enhancement. The controlled release mechanism operates through enzymatically responsive degradation or hydrolytic cleavage of polymer backbones, while surface functionalization techniques–including PEGylation and ligand conjugation–synergistically improve colloidal stability, prolong plasma residence time, and amplify pathological site recognition. Such multimodal design establishes polymeric nanoparticles as a next-generation platform for spatiotemporally controlled H_2_S delivery.^247^

The liposomal drug delivery system leverages the amphiphilic nature of phospholipids, which self-assemble into a bilayer vesicle structure. This allows simultaneous encapsulation of hydrophilic drugs in the aqueous core and hydrophobic drugs within the lipid membrane. The drug release mechanism of this nanocarrier primarily operates through dual pathways: Enzymatic degradation (phospholipase-mediated membrane phase transition) and passive diffusion driven by concentration gradients. Surface engineering modifications, particularly functionalization with targeting molecules (e.g., monoclonal antibodies, peptide ligands), significantly enhance the molecular recognition accuracy and targeted accumulation efficacy of liposomes in specific pathological microenvironments (such as tumor neovascular systems or inflammatory lesion areas). The successful development of this intelligent delivery system achieves spatiotemporal-controlled release of H_2_S molecules, providing an innovative solution for precision medicine.^247^

In the precision innovation of H_2_S therapeutic system, the research community has constructed multimodal intelligent controlled release platforms in recent years. Such systems have significantly enhanced the therapeutic index through the triple synergistic strategy of lesion-specific targeted delivery, homeostatic maintenance of release kinetics, and integrated monitoring of diagnosis and treatment. The research team led by Zhao developed an N-sulfhydryl-based controlled-release system, which not only achieved precise H_2_S release but also demonstrated significant cardioprotective effects in a murine MIRI model.^248^,^249^ It is worth noting that the ADT nanopores designed by the Takatani-Nakase research group demonstrated outstanding performance in an *in vitro* ischemia model: Compared with the traditional donor (NaHS) and metabolite ADT-OH, this delivery system not only achieved sustained release of H_2_S, but more importantly, showed remarkable efficacy in inhibiting cardiomyocyte apoptosis, highlighting the technical advantages of the new nanocarrier.^250^ Sun et al.^251^ developed a multifunctional nano-delivery system designated as DATS@MION-PEG-LF, which is constructed based on mesoporous iron oxide nanoparticles (MION) and exhibits therapeutic capabilities for targeted H_2_S delivery. This innovative system comprises four critical components: MION as the nanocarrier, the H_2_S donor DATS, a PEG surface modification layer, and the targeting ligand lactoferrin (LF). Through PEG functionalization, the researchers significantly enhanced the colloidal stability and blood circulation half-life of the nanoparticles. Concurrently, leveraging LF’s specific binding affinity with transferrin receptors on the BBB, the system achieved precise targeting of brain tissues. *In vitro* experiments demonstrated that this nanosystem could be efficiently internalized by neurons and cardiomyocytes. Under IRI conditions, the dual mechanisms of inhibiting the apoptotic pathway and scavenging ROS synergistically reduced OS-induced damage in both cerebral and myocardial tissues. Jiang et al.^252^ successfully developed an ultra-small platinum (Pt) sulfur cluster (Pt5.65S) pro-nanozyme system through the synergistic interaction between platinum cluster nanozymes and Na_2_S. This nanomaterial demonstrated exceptional OS regulatory capabilities in both RIRI and cisplatin-induced AKI animal models. Mechanistic studies revealed that the released H_2_S establishes a dual regulatory mechanism by mediating sulfhydration modification of NF-κB subunits while specifically inhibiting IκB kinase β activity. This synergistic action effectively blocked the cascade activation of NF-κB signaling pathway and inhibited its nuclear translocation, thereby suppressing inflammatory factor expression at the transcriptional level. Notably, owing to its ultra-small size characteristics, Pt5.65S can be efficiently cleared through glomerular filtration post-treatment, with *in vivo* experiments confirming absence of renal tissue accumulation, demonstrating ideal biosafety profiles. Zhang’s team innovatively utilized the covalent coupling property of 4-aminothiobenzamide with keratin to successfully construct a slow-release hydrogel system for H_2_S.^253^ The system realized the stable loading of H_2_S donor molecules through the covalent coupling strategy, which significantly prolonged the release cycle of the active gas, and its slow-release kinetic properties effectively improved the pharmacokinetic properties of H_2_S in organisms. The slow-release hydrogel prepared by the research team using the pluronic in the MIRI model, the F-127/keratin composite system exhibited significant cardioprotective effects. Experimental data demonstrated that this formulation reduces microvascular embolism area and mitigates myocardial fibrosis progression by suppressing neutrophil infiltration and downregulating the expression of key inflammatory factors (TNF-α and IL-6) in cardiac tissue. Further studies showed that this therapeutic effect originated from the targeted modulation of the NF-κB signaling pathway by the slow-release H_2_S, confirming the molecular mechanism of the gasotransmitter slow-release system in the treatment of cardiovascular diseases.

## Limitations

This study design also has several limitations. First, we only searched one database, so we inevitably missed out on the latest research developments. Second, we only reviewed previous studies and did not do any clinical trials or get clinical data to analyze and evaluate treatment effects. Also, there are still some challenges with using H_2_S in clinical settings, which is something worth looking into, and we can explore this further in the future.

## Conclusion and Perspectives

NO, CO, and H_2_S have attracted increasing attention as vital gasotransmitters in biological systems, playing pivotal roles in intercellular communication. These gaseous mediators not only share common biosynthetic origins but also exhibit remarkable functional convergence despite their distinct molecular mechanisms of action. Contemporary research highlights their paradoxical characteristics: While operating through divergent signaling pathways, these small-molecule transmitters demonstrate synergistic regulatory effects across diverse physiological and pathological contexts.

Emerging evidence positions H_2_S as a superior therapeutic candidate compared to conventional gasotransmitters like CO and NO, particularly in multimodal regulatory capacity and pharmacological applicability. At the molecular regulatory mechanism level, unlike the singular regulatory mechanisms of NO and CO, H_2_S exhibits multidimensional regulatory capabilities in pathological processes. Unlike single-target therapy, H_2_S simultaneously inhibits OS (via *Nrf2*/SOD activation), inflammation (via NLRP3 inflammasome inhibition and NF-κB down-regulation), and apoptosis (via regulation of the Bcl-2/Bax axis), forming a coordinated cytoprotective network. This multi-targeted action is essential to address the complex pathophysiology of IRI. For instance: By suppressing the production of inflammatory mediators like TNF-α, H_2_S significantly improves cerebral edema and reduces local inflammatory responses by blocking the NF-κB nuclear translocation pathway^61^,^254^; By mitigating cerebrovascular endothelial cell damage, inhibiting apoptosis, and regulating mitochondrial calcium homeostasis, H_2_S exerts neuroprotective effects against CIRI.^160^ In the field of gasotransmitter donor research, novel H_2_S donors demonstrate remarkable technical advantages. Representative agents like HSDF-NH_2_ exhibit optimized pharmacokinetic properties: Their sustained-release kinetic profile, exceptional BBB permeability, and proven safety profiles constitute distinct technical superiority.^193^ In contrast, NO donors suffer from poor chemical stability leading to uncontrolled release, with associated dose-dependent toxic side effects including systemic vasodilation and multi-organ hypoperfusion,^255^ significantly hindering their clinical translation prospects. CO-releasing molecules face bottleneck limitations such as metal ligand dissociation-induced cytotoxicity and low intracellular delivery efficiency.^256^ Furthermore, H_2_S has been extensively investigated across multiple medical domains including IS, acute MI, organ transplantation, neurodegenerative diseases, and oncology, it demonstrates more pronounced advantages over CO and NO in treating ischemic disorders, inflammatory conditions, and organ protection.

Over the past decade, the field of H_2_S research has witnessed revolutionary advancements. Early investigations predominantly focused on its cytotoxic effects at elevated concentrations. However, with the evolution of molecular biology technologies, the pleiotropic biological functions of H_2_S as a gaseous signaling molecule have been progressively uncovered. As a multi-organ system pathology, IRI has emerged as a critical clinical concern posing significant threats to human health. Substantial evidence confirms that both endogenous and exogenous H_2_S demonstrate multi-dimensional organ-protective effects in systemic IRI through mechanisms encompassing antioxidant stress responses, mitochondrial function modulation, and inflammatory regulation. Although this gaseous signaling molecule exhibits remarkable therapeutic potential across various disease models, the intricate molecular network underlying its actions in IRI remains enigmatic. This knowledge gap substantially impedes its clinical translation. The core challenge in this field lies in effectively translating fundamental research findings into clinical applications. Notably, the H_2_S research paradigm is undergoing a significant transformation, shifting emphasis from basic experimentation toward clinical implementation. Crucially, establishing a robust preclinical evaluation system–including safety assessments of novel H_2_S donors and optimization of administration protocols–remains imperative before advancing to human trials. Current clinical data on I/R therapeutics remains markedly insufficient, underscoring the need for deeper exploration into sulfur-containing compounds’ mechanisms across pathological microenvironments, particularly through molecular interactions and epigenetic regulation, to inform precision medicine strategies. Despite existing challenges, technological breakthroughs are reinvigorating H_2_S therapy development. Innovative approaches such as rationally designed sustained-release H_2_S donors and targeted nano-delivery systems show potential to enhance therapeutic specificity. Particularly noteworthy is the prospect of establishing synergistic therapeutic frameworks combining multiple gasotransmitters (CO and NO), coupled with advanced organ-specific I/R model analyses, which may pioneer novel H_2_S-based multimodal treatment paradigms. The convergence of interdisciplinary methodologies promises to illuminate the expanding role of H_2_S in IRI management, with its clinical application prospects warranting optimistic anticipation.

## Data Availability

*Not applicable*.
