# Corrigendum: Unlocking the gasotransmitter: hydrogen sulfide as a multitarget regulator in ischemia–reperfusion injury

**DOI:** 10.4103/mgr.MEDGASRES-D-26-00174

**Published:** 2026-07-23

In the article titled “Unlocking the gasotransmitter: hydrogen sulfide as a multitarget regulator in ischemia–reperfusion injury,” published on pages 221-240, Issue 3, Volume 16 of *Medical Gas Research* (doi: 10.4103/mgr.MEDGASRES-D-25-00100),^1^ the page number in the citation appears incorrectly. The correct page number is 221-240.

The correct citation is:

Chen Y, Deng Y, Tang R, Li S, Mei Z, Ge J. Unlocking the gasotransmitter: hydrogen sulfide as a multitarget regulator in ischemia–reperfusion injury. *Med Gas Res*. 2026;16(3):221-240.

## References

[1] Chen Y, Deng Y, Tang R, Li S, Mei Z, Ge J (2026). Unlocking the gasotransmitter: hydrogen sulfide as a multitarget regulator in ischemia-reperfusion injury. Med Gas Res.

